# Zoonotic and Anthroponotic *Plasmodium* spp. Circulation between Wild Primates and Indigenous Community, Peruvian Amazon, 2007–2020

**DOI:** 10.3201/eid3205.251695

**Published:** 2026-05

**Authors:** Gabriela M. Ulloa, Alex D. Greenwood, Omar E. Cornejo, Henar Alonso, Meddly L. Santolalla Robles, Stephanie Montero, Andres G. Lescano, Pedro Mayor

**Affiliations:** Universidade Federal Rural da Amazônia, Belém-Pará, Brazil (G.M. Ulloa); Universidad Científica del Sur, Lima, Peru (G.M. Ulloa); Leibniz-Institute for Zoo and Wildlife Research, Berlin, Germany (A.D. Greenwood); Freie Universität Berlin, Berlin (A.D. Greenwood); University of California Santa Cruz, Santa Cruz, California, USA (O.E. Cornejo); University of Zaragoza, Zaragoza, Spain (H. Alonso); Universidad Peruana Cayetano Heredia, Lima (M.L. Santolalla Robles, S. Montero, A.G. Lescano); Universidad Peruana de Ciencias Aplicadas, Lima (S. Montero); Universitat Autònoma de Barcelona, Bellaterra-Barcelona, Spain (P. Mayor); Comunidad de Manejo de Fauna Silvestre en la Amazonía y en Latinoamérica, Iquitos, Peru (P. Mayor); Museo de Culturas Indígenas Amazónicas, Iquitos (P. Mayor)

**Keywords:** malaria, zoonotic malaria, anthroponotic transmission, Plasmodium spp., nonhuman primates, Indigenous Peoples, Amazon rainforest, wildlife-human interface, cross-species pathogen flow, Peru

## Abstract

Malaria transmission at the human–wildlife interface remains poorly characterized in the Amazon. We conducted a molecular survey of *Plasmodium* spp. in an Indigenous community (n = 141) and sympatric nonhuman primates (NHPs) (n = 341; 10 species) in the Peruvian Amazon during 2007–2020. By using nested or quantitative PCR (targeting *cytb, cox3, and 18S rRNA genes*) and sequencing, we estimated prevalence, parasite load, and genetic similarity. We detected *Plasmodium* in 43.3% of humans and 51.9% of NHPs. *P. vivax*/*simium* predominated in humans (42.1%), whereas *P. brasilianum/malariae* predominated in NHPs (24.6%). *P. falciparum* was rare in both hosts. Children <8 years of age showed higher parasite load than older persons. Bayesian phylogenies revealed >99.9% identity among human and NHP lineages, supporting shared *Plasmodium* lineages. NHP lineages showed low interannual variation. One third of human infections were asymptomatic. Our findings reveal hidden reservoirs and support integrating wildlife surveillance into Amazon malaria elimination strategies.

Malaria parasites of nonhuman primates (NHPs) are increasingly recognized for their zoonotic potential and complicate malaria control efforts (*1*). The first report of a zoonotic malaria parasite capable of infecting humans was *Plasmodium knowlesi*, which emerged as a major cause of human malaria in Southeast Asia (*2*). In South America, *P. brasilianum* (morphologically, genetically, and immunologically indistinguishable from *P. malariae*) (*3*) and *P. simium* (closely related to *P. vivax*) (*4*) parasites have been detected in NHPs and implicated in zoonotic infections (*5*,*6*).

The Amazon basin harbors high biodiversity and dense human–wildlife overlap, particularly within Indigenous territories (*7*). Indigenous Peoples and local communities live in malaria-endemic areas with limited access to health systems and maintain close contact with wildlife (*8*), which creates favorable conditions for cross-species transmission of pathogens, including malaria parasites (*9*). This convergence of factors amplifies the risk for zoonotic and anthroponotic malaria transmission. Reports of natural infections with *P.* *vivax*, *P. simium*, *P. brasilianum*, *P. malariae*, and *P. falciparum* parasites in all neotropical NHP families underscore their potential reservoir role (*8*,*10*–*12*). Furthermore, experimental infections of neotropical NHPs with zoonotic malaria parasites from Asia raise concerns about the vulnerability of NHPs to emerging zoonoses (*13*).

Despite global declines in malaria incidence (*14*), progress in the Amazon is hindered by ecologic complexity, vector diversity, and high rates of subpatent infections (*15*). A recent study conducted in a remote community in the northeastern Peruvian Amazon reported a *P.* *vivax* prevalence of 56.0% based on quantitative PCR (qPCR) results; nearly half of the infections were submicroscopic, highlighting the persistence of hidden reservoirs despite ongoing control interventions (*16*). Subclinical and mixed infections, common in malaria-endemic areas, evade standard diagnostics and are rarely captured in surveillance systems (*17*). Despite this fact, malaria-control efforts remain focused on *P. falciparum* and *P.* *vivax* parasites, and little attention is given to NHP-associated species such as *P. brasilianum* or *P. malariae* (*18*).

Detecting hidden transmission dynamics requires highly sensitive, specific molecular tools and ethical field strategies (*19*,*20*). In remote forest settings, community-based methods, such as analyzing blood from legal subsistence hunting, can provide access to hard-to-reach wildlife populations while respecting Indigenous sovereignty (*21*). We investigated the molecular epidemiology of *Plasmodium* infections in sympatric humans and NHPs within a remote Indigenous territory in the Peruvian Amazon. By using blood samples from 141 Indigenous inhabitants and 341 neotropical NHPs (from 10 species) hunted for subsistence during an 11-year long-range wildlife collection program, we applied multigene molecular diagnostics and phylogenetic analysis to assess parasite prevalence, diversity, and evidence of cross-species transmission. 

## Methods

### Study Area

**Figure 1:**
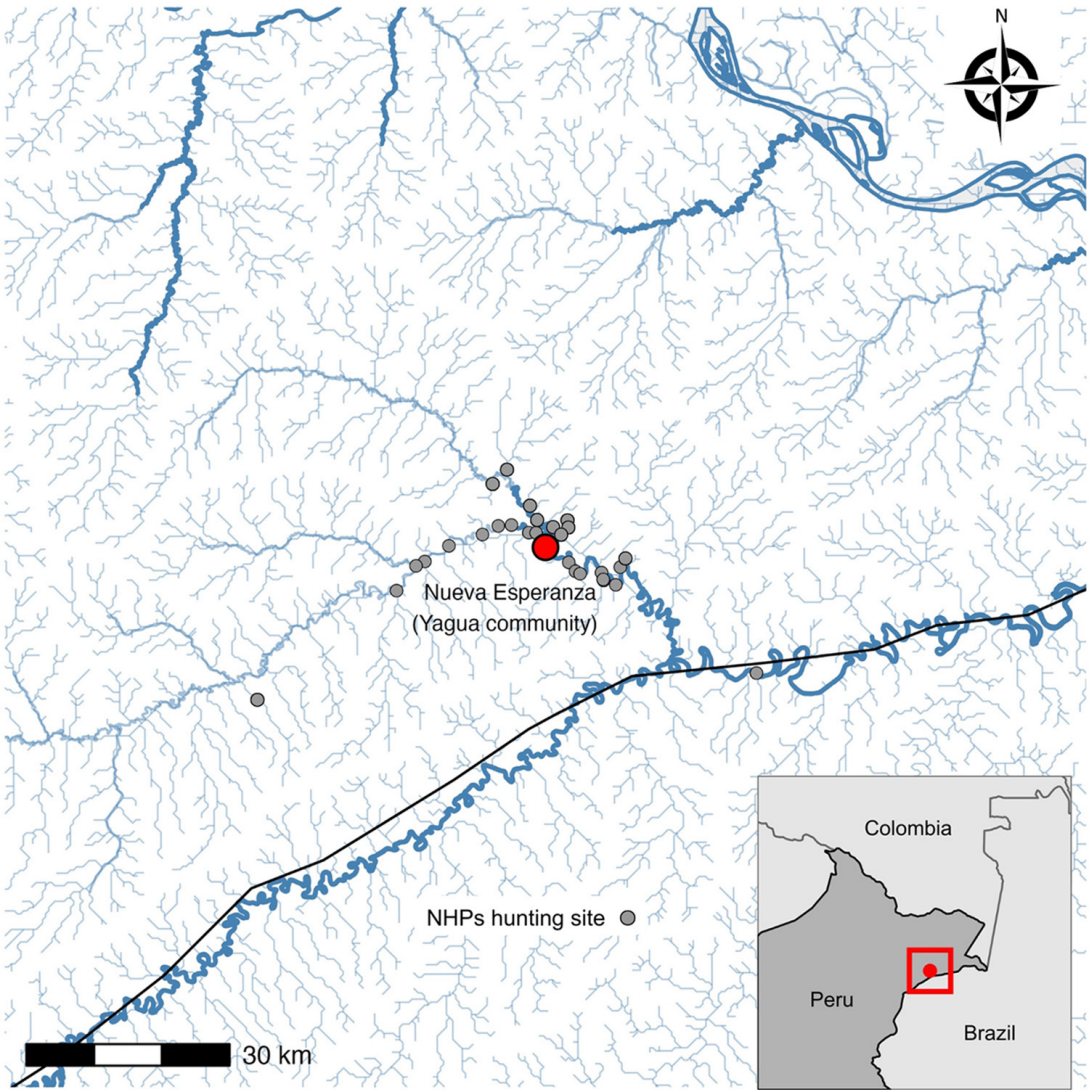
Geographic location of study of zoonotic and anthroponotic *Plasmodium* spp. circulation between wild primates and Indigenous community, Peruvian Amazon, 2007–2020. Red dot indicates Yagua Indigenous community of Nueva Esperanza, located along the Yavarí-Mirín River near the Peru–Brazil border. Gray dots represent locations of legally hunted nonhuman primates sampled during 2007–2020. Inset map indicates location of community (indicated by red dot in red square) at Peru–Brazil border.

We conducted this study in the Yavari-Mirin River basin, a remote upland forest region in the northeastern Peruvian Amazon. The area spans 107,000 hectares and includes a single Indigenous Yagua community, Nueva Esperanza, inhabited by 343 people in 2020. The community is located 302 km from the city of Iquitos and is surrounded by high biodiversity, including preserved populations of 14 NHP species (*22*). Malaria is endemic in the region; *P. vivax* is the most prevalent species. Villagers depend on subsistence activities (e.g., hunting, fishing, other forestry resources, and small-scale agriculture) and opportunistically trade wood, fish, wild meat, and agricultural products (Figure 1).

### Ethical Considerations

All human study procedures were approved by the Ethics Committees of Universidad Peruana Cayetano Heredia (approval no. 102142), Universitat Autònoma of Barcelona (Comisión de Ética en la Experimentación Animal y Humana approval no. 4829), and Hospital Clínic of Barcelona (approval no. HCB/2019/1107) and authorized by local and regional authorities of Nueva Esperanza (approval no. 267–2019-GRL-DRSL/30.09.01). NHP sampling was approved by the Peruvian Forest and Wildlife Service (approval no. 258–2019-MINAGRI-SERFOR-DGGSPFFS) and the Institutional Animal Use Ethics Committee of the Universidad Peruana Cayetano Heredia. We exported wildlife samples under Servicio Nacional Forestal y de Fauna Silvestre permits (nos. 003258/SP, 003260/SP, 003568-SERFOR, and 003579-SERFOR).

### Study Design and Blood Samples

In February 2020, we conducted a cross-sectional survey in Nueva Esperanza. We obtained whole blood samples through venipuncture from 141 participants (41.1% of the population) with informed consent, assent, or both. We referred participants with microscopic or clinical suspicion of malaria during the study to the community health center and treated them according to malaria management guidelines of Peru’s Ministry of Health (Supreme Decree no. 273–2025-MINSA).

In addition, we collected 17 samples from field workers; 5 were microscopically positive for *P. vivax* and had symptoms compatible with *P. vivax* infection; we molecularly analyzed samples of 3 of those workers and included results for sequence comparison only. Given the frequent malaria reports in Nueva Esperanza, field researchers received doxycycline as malaria chemoprophylaxis, in accordance with US Centers for Disease Control and Prevention (CDC) recommendations (*23*).

During 2007–2020, we collected dried blood spots (DBS) from 341 free-ranging NHPs (representing 10 species and 4 families) as part of a long-term wildlife monitoring program. Local hunters, trained in ethical sample collection, collected blood onto filter paper during postmortem handling of legally hunted animals. Species, sex, and date were recorded. The procedure was performed for all groups of mammals and birds (not only for wild NHPs) to avoid encouraging hunting of NHPs. The species sampled included 143 Poeppig’s woolly monkeys (*Lagothrix lagothrica poeppigii*), 57 large-headed capuchin monkeys (*Sapajus macrocephalus*), 43 black spider monkeys (*Ateles chamek*), 29 Ucayali bald uakaris (*Cacajao calvus ucayalii*), 21 red howler monkeys (*Alouatta seniculus*), 19 Humboldt’s white-fronted capuchin monkeys (*Cebus albifrons*), 16 monk saki (*Pithecia monachus*), 8 Ecuadorian squirrel monkeys (*Saimiri macrodon*), 4 coppery titi monkeys (*Plecturocebus cupreus*), and 1 brown-mantled tamarin (*Leontocebus fuscicollis*).

### Microscopic Examination

We examined thick blood smears for 135 participants; 10 were positive and all confirmed by molecular assays. Because of low positivity and variable slide quality, we excluded microscopic examination results from analyses.

### Molecular Diagnosis of *Plasmodium* Species

We extracted DNA from whole blood and DBS by using the AllPrep DNA/RNA Mini Kit (QIAGEN, https://www.qiagen.com). We collected all DBS from NHP in remote field conditions during 2007–2020, often with low DNA concentration and partial degradation. Therefore, we selected molecular targets to maximize sensitivity in cases of low parasitaemia and degraded material.

We detected parasites by using 2 nested PCRs and 2 commercial VIASURE qPCR diagnostic kits (Certest Biotec, https://www.certest.es) targeting mitochondrial cytochrome oxidase b (*cytb*), cytochrome c oxidase III (*cox3*), and *18S SSU rRNA* genes. For quantitative analyses, we generated a standard curve by using serial dilutions of the kit’s Malaria Quantitative Standard (≈2 × 10^7^ copies/µL) to estimate genome copy number. We converted cycle threshold values to DNA copies/µL and log_10_-transformed for statistical comparisons. Because of gene *18S rRNA* copy number variability among *Plasmodium* species, our estimates reflect relative (not absolute) parasitemia.

### Partial Amplification of *cytb* Gene

We performed nested PCR (nPCR) for *cytb* to detect *Plasmodium* spp. parasites in both humans and NHP samples, as described previously (*24*). PCRs included appropriate positive and negative controls and were processed under protocols that prevented contamination. We sequenced amplicons for species confirmation.

### Validation of *Plasmodium* spp. Detection

**Figure 2:**
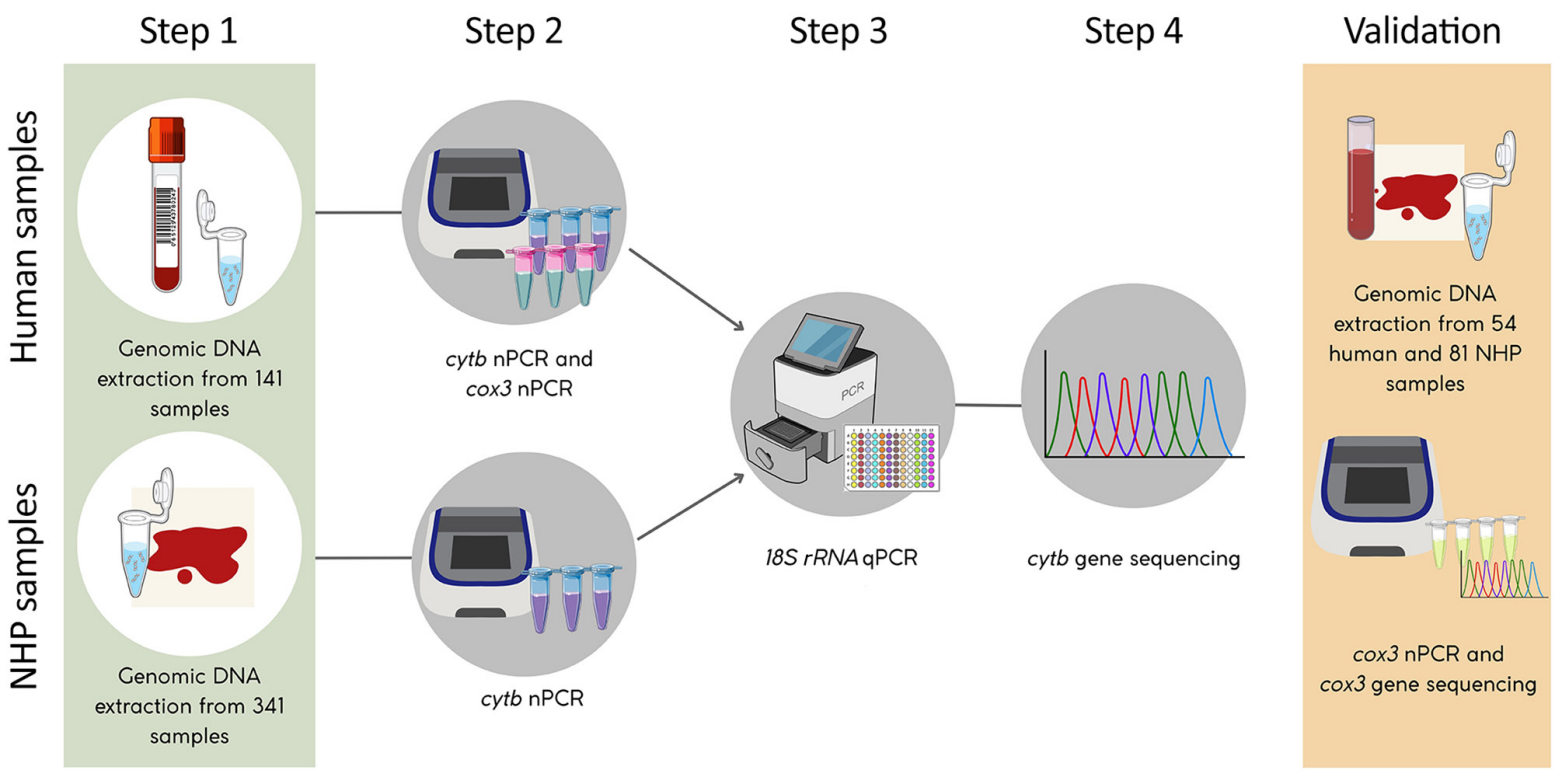
Workflow for molecular detection and validation of *Plasmodium* spp. infections in study of zoonotic and anthroponotic *Plasmodium* spp. circulation between wild primates and Indigenous community, Peruvian Amazon, 2007–2020. Schematic of laboratory procedures show DNA extraction and detection of *Plasmodium* spp. in human and NHP blood samples using qPCR (targeting *18S rRNA* gene) and nPCR targeting mitochondrial genes (*cytb* and *cox3*). Selected samples underwent sequencing for species confirmation and external validation. *cytb*, cytochrome oxidase b; *cox3*, cytochrome c oxidase III; NHP, nonhuman primate; nPCR, nested PCR; qPCR, quantitative PCR.

We validated a subset of 54 human and 81 NHPs samples at Leibniz Institute for Zoo and Wildlife Research (Berlin, Germany) (Figure 2) by using *cox3* nPCR and sequencing. The *cox3* nPCR uses genus-specific primers for the primary PCR, whereas the secondary PCR uses species-specific primers. The genus-specific primers were designed to avoid regions of high sequence similarity to human mitochondrial DNA, and each species-specific primer pair differs by >7 nucleotides at the 3′ end, which is critical for specificity. We used the oligonucleotides 5′-CTC GCC ATT TGA TAG CGG TTA ACC-3′ (forward) and 5′-CCT GTT ATC CCC GGC GAA CCT TC-3′ (reverse) as primers, as previously described (*25*), with some modifications: we performed first-round PCR in 25-μL reactions with 2 μM of each primer, 12.5 μL 2X MyTaq Mix (Meridian Bioscience, https://www.meridianbioscience.com), and 5 μL of template DNA. We conducted thermocycling: 96°C for 1 minute, followed by 40 cycles of 96°C for 10 seconds, 63°C for 1 minute, 72°C for 1 minute, and a final extension at 72°C for 10 minutes.

Nested reactions used 2.5 μL of 1:50-diluted PCR product, 4 μM primers, and 10 μL of 2X MyTaq Mix. We conducted cycling on *P. falciparum* and *P. vivax*/*P. simium* samples: 96°C for 1 minute; 30 cycles of 96°C for 10 seconds, 54°C for 1.5 minute, 72°C for 1 minute; and final extension at 72°C for 10 minutes. We applied the same protocol for *P. brasilianum*/*P. malariae* with 58 °C annealing. We gel-purified and sequenced amplicons for species confirmation. We performed nPCR separately for *P. falciparum*, *P. vivax*/*P. simium*, and *P. brasilianum*/*P. malariae* to generate sequences (Appendix Table 1).

### Statistical Analysis

We analyzed data in R version 4.4.2 (The R Project for Statistical Computing, https://www.r-project.org). We estimated associations between symptoms and *Plasmodium* infection by using prevalence ratios and 95% CIs. We inferred parasite load from cycle threshold values converted to genome copy number (DNA copies/μL) by using a standard curve based on *Plasmodium 18S rRNA* qPCR and expressed these values as log_10_(DNA/μL + 1).

We assessed differences by age (<8 years vs. >8 years) and symptom status using Welch’s t-tests or Wilcoxon tests. We used linear regression models (Gaussian family, identity link) to evaluate age–symptom interactions.

We analyzed interannual NHPs prevalence by using a generalized linear model (binomial family, logit link) with host species and year as predictors. We assessed temporal stability by using coefficients of variation (CV), which we calculated as the SD divided by the mean prevalence per host–parasite pair; lower CV values indicated more stable infection patterns.

We compared patristic distances by using Kruskal-Wallis test and used pairwise Wilcoxon tests with Holm correction for multiple comparisons. We evaluated diagnostic agreement by using positive percent agreement (PPA), negative percent agreement (NPA), Cohen kappa coefficient, and percent concordance, using *cytb* nPCR sequencing as the reference standard. We defined statistical significance at α = 0.05.

### Phylogenetic Analysis

We manually inspected chromatograms and corrected them for base-calling errors. We assembled consensus sequences in Geneious Prime version 2025.0.3 (Geneious, https://www.geneious.com) (*26*). We performed multiple sequence alignment in Geneious using the Clustal-Omega algorithm and visually trimmed ambiguous regions, resulting in final alignments of ≈776 bp of the mitochondrial *cytb* gene.

We inferred preliminary phylogenies by using the neighbor-joining method with 1,000 bootstrap replicates. We selected the best-fit nucleotide substitution model according to the Akaike information criterion in MEGA version 11.0.13 (*27*). We reconstructed final trees by using maximum-likelihood in FastTree version 2.1.11 (*28*) and Bayesian inference in MrBayes version 3.2.6 (*29*), applying the selected substitution model in both. Bayesian inference analyses consisted of 2 independent runs of 3 million generations, sampling every 1,000 generations, and a 25% burn-in.

We included reference *Plasmodium* sequences from GenBank and PlasmoDB to contextualize the evolutionary placement of lineages detected in humans and NHPs. Those sequences were *P. falciparum*, *P. vivax* (Sal-I, P01, PAM), *P. simium*, *P. malariae*, *P. brasilianum*, and rodent (*P*. *chabaudi*, *P. vinckei*, *P. berghei*, and *P. yoelii*) and avian (*P. gallinaceum* and *P. relictum*) *Plasmodium* species. The Apicomplexa parasite *Toxoplasma gondii* served as the outgroup. In addition, we incorporated 3 sequences from symptomatic field researchers, confirmed as *P. vivax* infections by microscopic examination and qPCR to assess their phylogenetic relationship with community-derived human and NHP infections.

### Data Availability 

Anonymized metadata will be available upon reasonable request. Sequence data are in GenBank (accession nos. PV769906–68 and PV786447–592) (Appendix Table 2). Protocols are available at https://www.protocols.io (Appendix).

## Results

### Prevalence of *Plasmodium* spp. in Humans and NHPs

Among 141 human participants, we detected *Plasmodium* parasites in 35.5% (50/141) by qPCR and in 43.3% (61/141) by *cytb* nPCR. In 341 NHPs, prevalence reached 31.2% (69/221) by qPCR) and 51.9% (177/341) by *cytb* nPCR. Species-specific qPCR revealed *P. vivax*/*P. simium* in 30.5% (43/141) of samples, including 2 *P. falciparum* co-infections (1.4% [2/141]). Five samples were positive only at genus-level by qPCR. *cytb* nPCR sequencing confirmed *P. vivax*/*P. simium* in 42.1% (58/141) of participants, with single cases of *P. falciparum* and *P. brasilianum*/*P. malariae* (0.7% [1/141 each]). Species-level identification was successful in 98.4% (60/61) of nPCR-positive samples.

Among 110 persons for whom data were complete, 38 (34.6%) reported malaria-like symptoms (e.g., fever, headache, vomiting, nausea, pallor, or diarrhea). Of qPCR-positive patients, 16 (37.2%) were asymptomatic. Symptomatic persons were 3.8 times more likely to test positive (prevalence ratio 3.82 [95% CI 2.13–6.88]) (Table 1).

**Table 1:** Association between clinical symptoms and *Plasmodium* spp. quantitative PCR results in 110 members of Indigenous Yagua community, Nueva Esperanza, Loreto region, Peru, 2020*

Result	No. (%) asymptomatic persons, n = 72	No. (%) symptomatic persons, n = 38†	Prevalence ratio (95% CI)‡	p value§
Negative	56 (77.8)	11 (28.9)	Referent	<0.001
Positive	16 (22.2)	27 (71.1)	3.82 (2.13–6.88)	

*Prevalence ratios and 95% CIs estimated by using the cohort.count method in epiR (The R Project for Statistical Computing, https://www.r-project.org). †Defined as persons reporting >1 malaria-related symptom (e.g., fever, headache, vomiting, nausea, pallor, or diarrhea) at the time of sampling. ‡Calculated by using asymptomatic persons as reference. §By Fisher exact test.

In NHPs, qPCR detected *P. brasilianum*/*P. malariae* in 25.8% (57/221), *P. vivax*/*P. simium* in 3.2% (7/221), and *P. falciparum* in 0.5% (1/221). *cytb* nPCR sequencing confirmed *P. brasilianum*/*P. malariae* in 24.6% (84/341), *P. vivax*/*P. simium* in 17.9% (61/341), and *P. falciparum* in 0.3% (1/341) (Table 2). Sequencing success was 82.5% (146/177).

**Table 2:** Prevalence of *Plasmodium* spp. detected by nested PCR targeting *cytb* gene in 341 nonhuman primates from 10 species sampled in Yavarí-Mirín River basin, Loreto region, Peru, 2007–2020*

Host species	Total no. samples	*Plasmodium* spp., no. (%)	*P. brasilianum* or *P. malariae*, no. (%)	*P. vivax* or *P. simium*, no. (%)	*P. falciparum*, no (%)
Atelidae	207	92 (44.44)	43 (22.75)	31 (16.40)	0
* Lagothrix lagothrica poeppigii*	143	57 (39.86)	32 (24.24)	14 (10.61)	0
* Ateles chamek*	43	18 (41.86)	5 (12.82)	9 (23.08)	0
* Alouatta seniculus*	21	17 (80.95)	6 (33.33)	8 (44.44)	0
Cebidae	84	48 (57.14)	15 (20.27)	23 (31.08)	0
* Sapajus macrocephalus*	57	26 (45.61)	5 (9.80)	15 (29.41)	0
* Cebus albifrons*	19	14 (73.68)	7 (41.18)	5 (29.41)	0
* Saimiri macrodon*	8	8 (100)	3 (50.00)	3 (50.00)	0
Pitheciidae	49	36 (73.47)	24 (53.33)	7 (15.56)	1 (2.22)
* Cacajao calvus ucayalii*	29	22 (75.86)	18 (66.67)	2 (7.41)	0
* Pithecia monachus*	16	10 (62.50)	6 (40.00)	2 (13.33)	1 (6.67)
* Plecturocebus cupreus*	4	4 (100)	1 (25.00)	3 (75.00)	0
Callitrichidae	1	1 (100)	1 (100)	0	0
* Leontocebus fuscicollis*	1	1 (100)	1 (100)	0	0
Total	341	177 (51.91)	84 (24.63)	61 (17.89)	1 (0.29)

*Data presented by taxonomic group and species. Percentages reflect proportion of positive persons for each parasite species within sampled group. Prevalence values calculated relative to total persons per host species.

**Figure 3:**
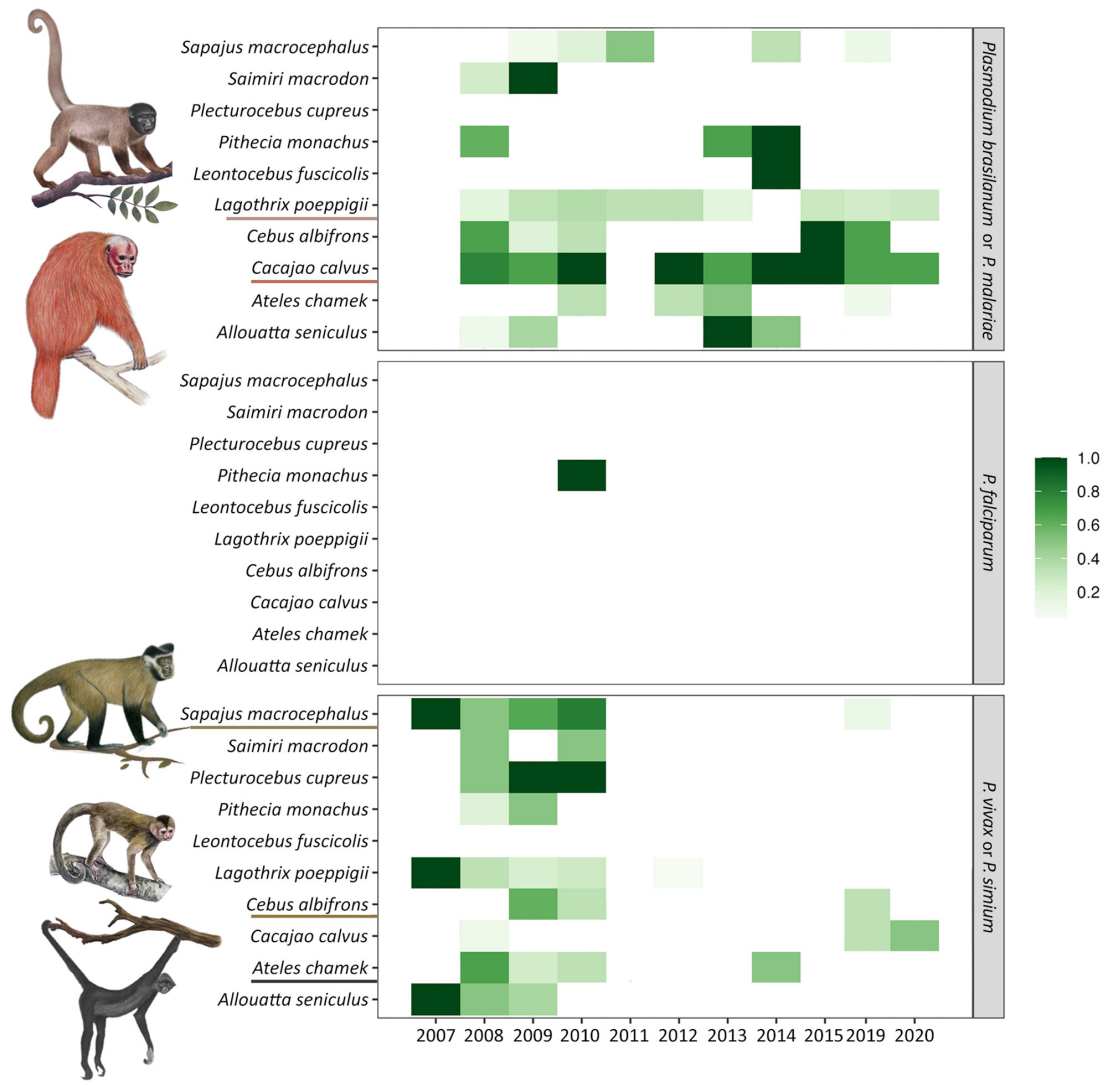
Annual prevalence of *Plasmodium* spp. infections in wild nonhuman primate species detected in study of zoonotic and anthroponotic *Plasmodium* spp. circulation between wild primates and Indigenous community, Peruvian Amazon, 2007–2020. Prevalence of cytochrome oxidase b nested PCR–detected infections are shown for 10 nonhuman primate species sampled during 2007–2015, 2019, and 2020 in the Yavarí-Mirín River basin, Loreto region, Peru. Each tile indicates the proportion of infected persons for a given host–parasite pair, by year (shading intensity reflects prevalence). Illustrations and underlining highlight taxa with more stable or recurrent infection patterns.

We observed no statistically significant interannual trend for *P. brasilianum*/*P. malariae* in NHPs (p = 0.414). However, host-specific variation emerged. Ucayali bald uakaris showed low interannual variation (CV 0.20, n = 29), suggesting stable *P. brasilianum*/*P. malariae* transmission. Poeppig’s woolly monkeys also showed sustained *P. brasilianum*/*P. malariae* prevalence (CV 0.26, n = 143). For *P. vivax*/*P. simium*, Humboldt’s white-fronted capuchin monkeys had the most consistent pattern (CV 0.36, n = 19), whereas black spider monkeys and large-headed capuchin monkeys showed more fluctuation (Figure 3; Appendix Table 3).

### Parasite Load

**Figure 4:**
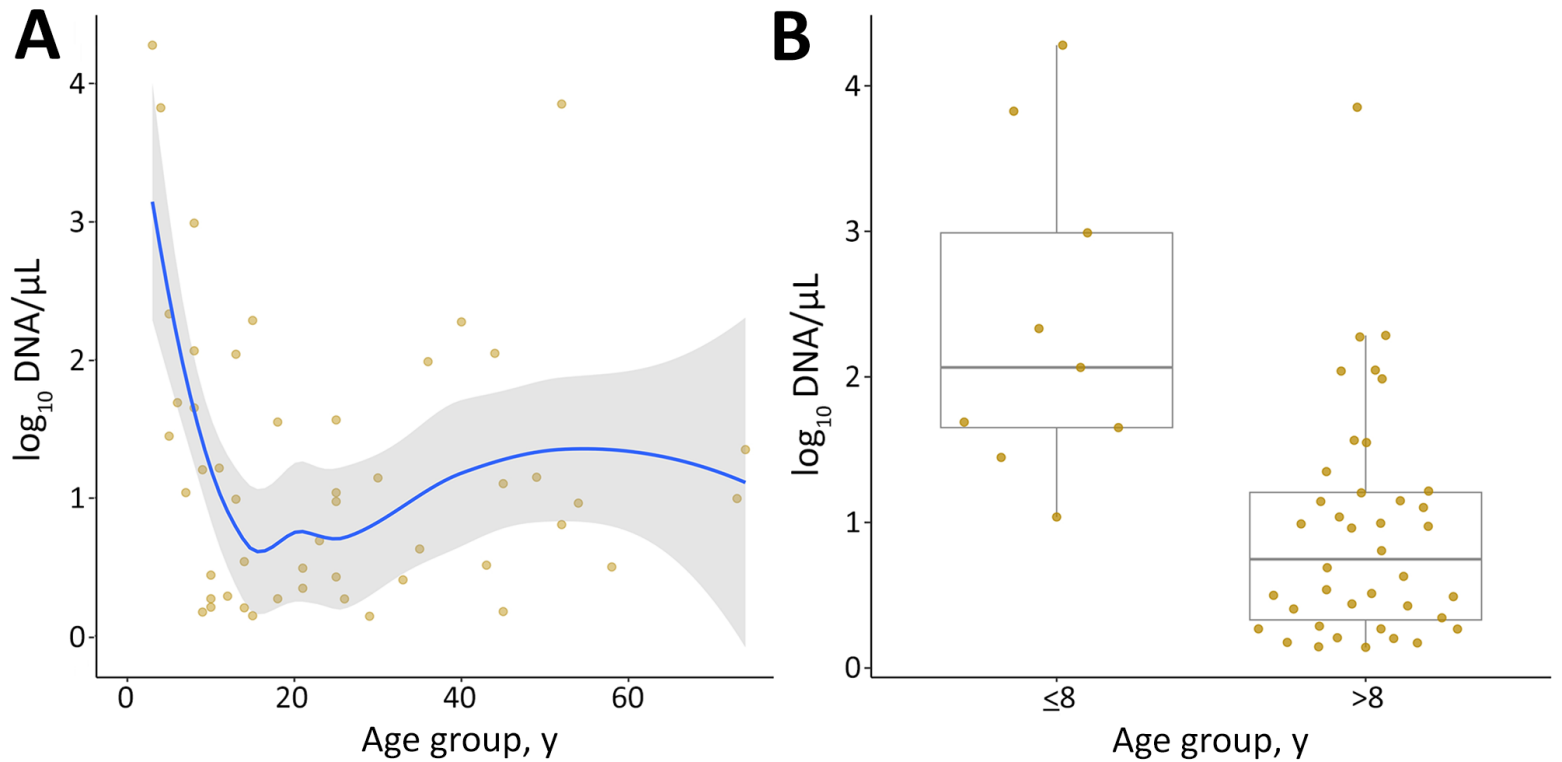
Age-specific parasite load in humans based on quantitative PCR targeting for *Plasmodium* spp. *18S rRNA* genes in study of zoonotic and anthroponotic *Plasmodium* spp. circulation between wild primates and Indigenous community, Peruvian Amazon, 2007–2020. A) Smoothed regression of log-transformed parasite DNA concentration, by age, in 49 persons. B) Boxplot comparing parasite loads between children <8 years and older persons. Higher parasite loads occurred in younger age groups. Horizontal lines within boxes indicate medians; box tops and bottoms indicate interquartile range (25th–75th percentiles); error bars indicate 1.5 × interquartile range.

Participants (n = 141) included 84 women (59.6%) and 57 men (40.4%). Median age was 21 years (interquartile range 11–35, range 3–79 years); 9 participants (6.4%) were <8 years of age. Children <8 years of age had significantly higher relative parasite DNA levels inferred from qPCR (mean +SD log_10_ 2.4 +1.1 vs. 0.9 +0.8; p = 0.005) than older persons (Figure 4). We found no significant differences in parasite load between symptomatic and asymptomatic participants. Linear modeling showed higher relative parasite loads in young children (β = 1.28, p = 0.020), independent of symptoms. We found no interaction between age and symptom status (p = 0.750).

**Figure 5:**
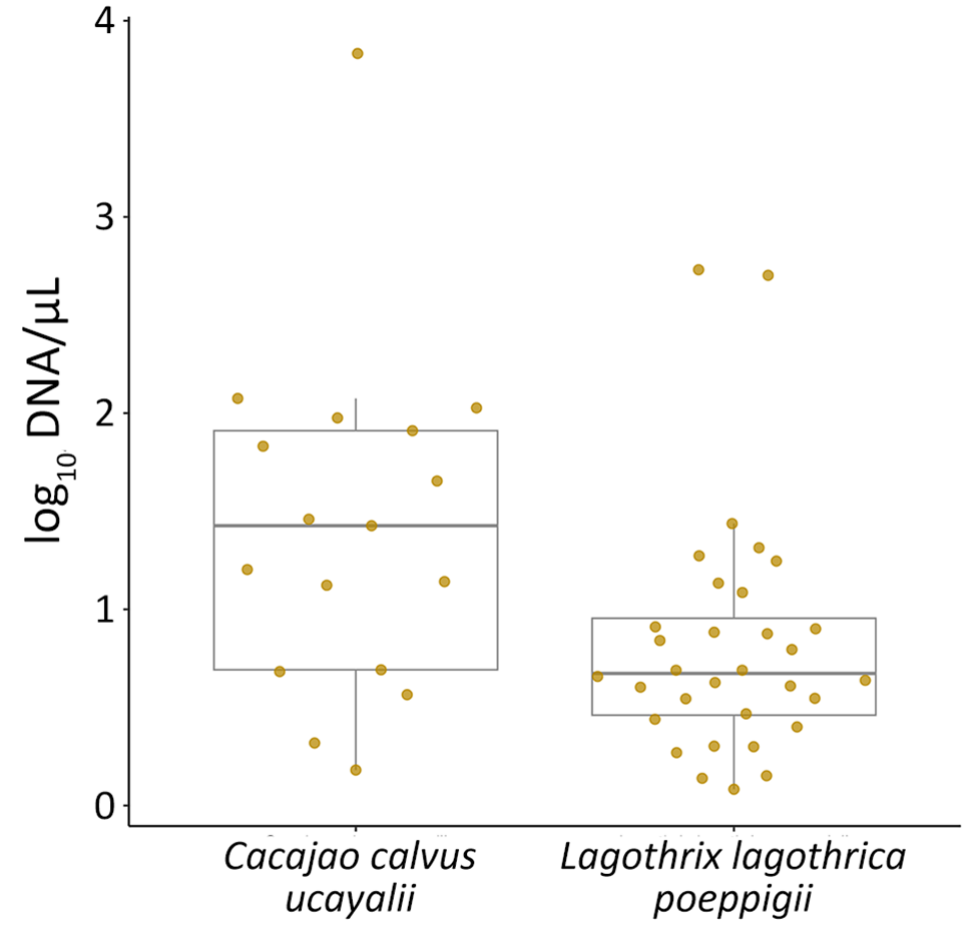
Comparison of parasite loads in 2 nonhuman primate species with *Plasmodium brasilianum* or *P. malariae* infections in study of zoonotic and anthroponotic *Plasmodium* spp. circulation between wild primates and Indigenous community, Peruvian Amazon, 2007–2020. Relationship boxplots of log-transformed *P. brasilianum* or *P. malariae* DNA concentration in 17 Ucayali bald uakaris (*Cacajao calvus ucayalii*) and 31 Poeppig’s woolly monkeys (*Lagothrix lagothrica poeppigii*), highlighting species-specific differences in parasitaemia. Horizontal lines within boxes indicate medians; box tops and bottoms indicate interquartile range (25th–75th percentiles); error bars indicate 1.5 × interquartile range.

In NHPs, *P. brasilianum*/*P. malariae* infections had higher relative parasite DNA levels than *P. vivax*/*P. simium* (1.2 + 0.8 vs. 0.6 + 0.3; p<0.001). *P. vivax* in humans showed higher relative loads than *P. vivax*/*P. simium* in NHPs (p = 0.044), whereas *P. vivax* in humans and *P. brasilianum*/*P. malariae* in NHPs had similar densities (p = 0.851) (Table 3; Appendix Figure 2). Among NHPs, Ucayali bald uakaris exhibited significantly higher *P. brasilianum*/*P. malariae* relative loads than did Poeppig’s woolly monkeys (1.4 +0.9 vs. 0.8 +0.6; p = 0.020) (Figure 5).

**Table 3:** Comparison of parasite load, by host and *Plasmodium* species, based on quantitative PCR targeting *18S rRNA* gene in humans and NHPs from the Yavarí-Mirín River basin, Loreto region, Peru*

Host	Parasite species	log_10_ DNA/μL, mean +SD	Comparison	p value
NHP, n = 57	*P. brasilianum*/*P. malariae*	1.2 +0.8	vs. *P. vivax*/*P. simium* (NHP)	0.023
NHP, n =7	*P. vivax*/*P. simium*	0.6 +0.3	vs. *P. vivax*/*P. simium* (human)	0.044
Human, n = 43	*P. vivax*/*P. simium*	1.2 +1.0	vs. *P. brasilianum*/*P. malariae* (NHP)	0.851

*Parasite load expressed as log_10_-transformed DNA concentration per μL. Statistical comparisons performed using pairwise tests between groups. NHP, nonhuman primate.

### Sequence and Phylogenetic Analysis

We analyzed a total of 209 partial *cytb* sequences, consisting of 60 human sequences, 3 sequences from fieldworkers, and 146 sequences from NHPs. Intraspecific and interspecific patristic distances based on *cytb* sequences revealed marked differences in genetic divergence among *Plasmodium* species across host groups (χ^2^ = 11.18 by Kruskal-Wallis test; degrees of freedom = 4, p<0.001). Human sequences had lower genetic divergence (mean 0.007) than did NHPs (mean 0.03; p<0.001). *P. vivax*/*P. simium* sequences from both hosts had very low divergence (mean 0.0007); *P. brasilianum*/*P. malariae* sequences showed even lower divergence (mean 0.0005, p<0.001). We found *P. falciparum* in 1 human and 1 NHP, with 99.6% identity.

**Figure 6:**
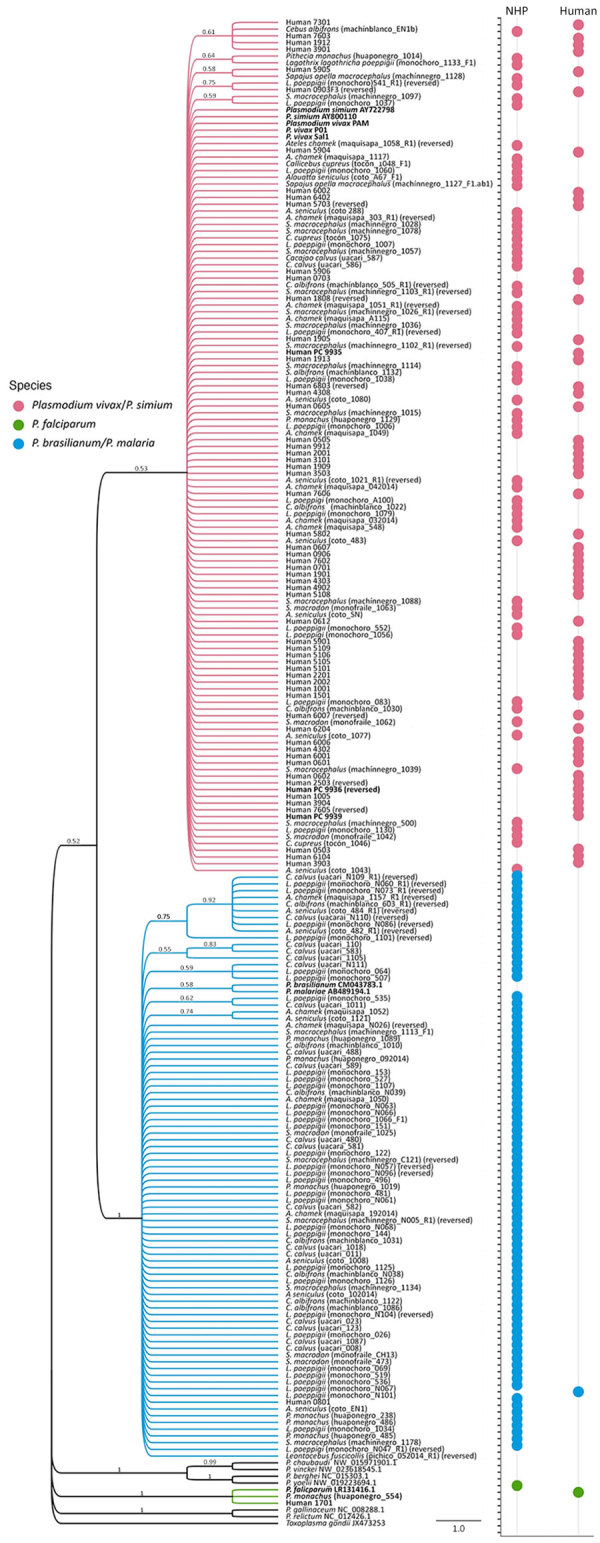
Bayesian phylogeny of *Plasmodium* spp. from humans and nonhuman primates in study of zoonotic and anthroponotic *Plasmodium* spp. circulation between wild primates and Indigenous community, Peruvian Amazon, 2007–2020. Phylogenetic tree shows cytochrome oxidase b gene sequences (≈776 bp) obtained from 60 humans and 146 wild nonhuman primates sampled in the Yavarí-Mirín River basin, Loreto region, Peru. Tree reconstructed by using Bayesian inference (209 sequences). Tip symbols denote host origin (human or nonhuman primates) and are color-coded by species: pink, *P. vivax* or *P. simium*; blue, *P. brasilianum* or *P. malariae*; and green, *P. falciparum*; black, reference sequences. Bold type indicates sequences from fieldworkers with confirmed *P. vivax* infection.

Bayesian phylogenetics revealed 3 clades: *P. vivax*/*P. simium*, *P. brasilianum*/*P. malariae*, and *P. falciparum*. Most (96.7% [58/60]) human sequences and 41.8% (61/146) of NHP sequences clustered within the *P. vivax*/*P. simium* clade (Figure 6). All fieldworkers’ sequences also clustered within *P. vivax*/*P. simium*. Sequences clustering within the *P. brasilianum*/*P. malariae*clade comprised 57.5% (84/146) of NHP and 1.7% (1/60) of human sequences. *P. falciparum* appeared in 1 human and 1 NHP. FastTree and neighbor-joining trees matched the Bayesian results, showing consistent clustering and no host-specific structuring (Appendix Figures 3, 4).

### Validation of *Plasmodium* Detection

We reanalyzed a subset of 54 human and 81 NHP samples at Leibniz Institute for Zoo and Wildlife Research by using *cox3* nPCR. In humans, we confirmed *P. vivax*/*P. simium* in 31.5% (17/54) and mixed infections in 11.1% (6/54). Laboratory concordance was 83.3% (kappa 0.66). In NHPs, we confirmed *P. brasilianum*/*P. malariae* in 19.8% (16/81) and *P. vivax*/*P. simium* in 16.1% (13/81). Agreement metrics were as follows: PPA 25.4%, NPA 95.2%, kappa 0.08. For *P. brasilianum*/*P. malariae* only, agreement metrics were PPA 44.0%, NPA 100.0%, and kappa 0.40. We consistently recovered major parasite lineages across laboratories and markers, indicating the robustness of detecting dominant parasite lineages despite low interlaboratory concordance for wildlife samples attributable to low DNA concentration and partial degradation (Appendix Figure 5).

## Discussion

The challenge facing infectious disease studies in free-ranging wild animals lies in the accessibility of biological material. Therefore, we implemented a culturally sensitive and sustainable surveillance approach on the basis of blood samples from discarded byproducts of legal subsistence hunting by Indigenous Peoples, respecting national laws and local practices and enabling long-term access to remote, well-preserved ecosystems, regions that are typically underrepresented in public health and zoonotic research (*20*).

Our results revealed a high prevalence of *Plasmodium* spp. in both humans and NHPs. *P. vivax* and *P. simium* were dominant in humans, whereas *P. brasilianum* and *P. malariae* predominated in NHPs and included sporadic *P. falciparum* co-infections. Parasite load decreased with age in humans, suggesting development of partial immunity from around 8 years of age, consistent with previous evidence of early adaptive immune response to *P. vivax* (*30*).

Nonetheless, one third of infections were asymptomatic, highlighting the role of subclinical carriage in sustaining silent transmission (*31*). In addition, similar findings in the remote community of Santa Emilia, in the northeastern Peruvian Amazon, indicated 44.0% submicroscopic *P. vivax* infections, underscoring the widespread presence of low-parasitemia reservoirs across the Peruvian Amazon (*16*). However, our study extends this evidence to sympatric wild primates, revealing a broader eco-epidemiologic interface of *Plasmodium* spp. circulation.

Assay performance varied by host and sample type, particularly in cases of low parasitaemia and long-term stored DBS from NHPs. Differences between qPCR (targeting the *18S rRNA* gene) and nPCR (targeting the *cytb* gene) probably reflect gene copy number variation (*32*). Incorporating an independent *cox3* nPCR improved confirmation of infection and detection of coinfections, enhancing resolution for subpatent infections in both hosts. However, large-scale implementation remains challenging because of structural barriers, limited access to healthcare, and reliance on suboptimal diagnostic tools, which contribute to underreporting of malaria cases and are critical obstacles to effective surveillance and control (*33*).

Molecular and phylogenetic analyses confirmed *P. vivax*/*P. simium* and *P. brasilianum*/*P. malariae* in both hosts. Although human sampling occurred in 2020 and NHP samples spanned 2007–2020, several human sequences matched NHP sequences collected more than a decade earlier, suggesting long-term maintenance of shared *Plasmodium* lineages across hosts, rather than recent spillover. Human *P. vivax*/*P. simium* sequences showed high mitochondrial similarity, consistent localized outbreaks, whereas greater diversity in NHPs indicates their role as long-term reservoirs.

*Plasmodium* spp. prevalence in NHPs ranged from 33% to 52% and exhibited species-specific variation. Ucayali bald uakaris (*C. c. ucayalii*), Poeppig’s woolly monkeys (*L. l. poeppigii*), Humboldt’s white-fronted capuchin monkeys (*C. albifrons*), black spider monkeys (*A. chamek*), and large-headed capuchin monkeys (*S. macrocephalus*) showed higher relative parasite DNA levels and stable infections, potentially reflecting differences in host susceptibility, immune tolerance, ecologic exposure, and evolutionary history (*34*). Low-grade chronic infections in NHPs might parallel asymptomatic infections in humans (*35*). Of note, the uakari (*C. c. ucayalii*) of Peru is classified as a vulnerable species because of its restricted distribution (*36*). This species has distinctive red facial vasculature and shows consistently higher relative parasitaemia than animals from sympatric taxa (*37*). Although the biologic basis of this pattern remains unclear, it may reflect species-specific ecologic or physiologic traits rather than host–parasite coevolution.

The single *P. falciparum* detection in a wild NHP (monk saki [*P. monachus*]), confirmed by 2 independent molecular assays, probably represents anthroponotic spillover. This finding underscores the permeability between human and animal malaria cycles and the need to monitor bidirectional transmission (*10*,*11*).

Our study provides longitudinal molecular evidence of *Plasmodium* spp. circulation in wild Amazonian NHPs (from 10 species) and 341 humans over an 11-year period. This approach, which integrated wildlife and human screening in parallel, provided a unique ecologic and temporal view of malaria dynamics in a remote Indigenous territory. Endemic *P. brasilianum*/*P. malariae* maintenance in wildlife contrasts with sporadic, low-parasitaemia *P. vivax*/*P. simium* detection. This pattern might reflect a complex eco-immunologic balance (*34*,*35*) in which *P. vivax*/*P. simium* circulates at undetectable levels in adapted NHP hosts, whereas *P. brasilianum*/*P. malariae* persists more overtly. Relative parasite DNA levels inferred from qPCR in *P. vivax*/*P. simium*–infected NHPs were ≈4 times lower than in local humans with low parasitaemia, reinforcing the need for sensitive and specific surveillance tools to detect cryptic reservoirs. However, that result should be interpreted cautiously, given that molecular estimates do not replace measurements acquired through microscopic examination.

Our findings carry important implications for malaria elimination. In Amazonian contexts, where humans and wildlife are closely linked through ecology and culture, traditional surveillance might miss important sources of transmission. Asymptomatic human infections and cryptic wildlife reservoirs add complexity to malaria ecology, reinforcing the need for One Health approaches that integrate wildlife monitoring and sensitive diagnostics.

One limitation of our study is that NHP samples were obtained opportunistically, taking advantage of waste materials from subsistence hunting; therefore, species representation reflects ecologic, behavioral, and seasonal factors, as well as hunter preferences influencing hunting practices, rather than the full primate community (*38*). Prevalence estimates thus should be interpreted carefully. We inferred parasite load from molecular data, and relative parasite DNA levels derived from qPCR should be interpreted cautiously because they do not replace parasitaemia measurements acquired through microscopic examination. We selected mitochondrial markers to optimize amplification from long-term storage DBS, low-parasitaemia samples, prioritizing sensitivity over fine-scale taxonomic resolution, but their limited resolution constrains discrimination between closely related parasites such as *P. vivax* and *P. simium*.

In conclusion, this study demonstrates that *Plasmodium* spp. transmission in the Peruvian Amazon involves overlapping human and NHPs reservoirs and that distinct parasite species predominate in each host. We show high prevalence of *P. vivax*/*P. simium* in humans and sustained circulation of *P. brasilianum*/*P. malariae* across multiple NHP species over more than a decade, confirming long-term maintenance of malaria parasites in wildlife. Detection of asymptomatic human infections and low-parasitaemia infections in NHPs highlights a hidden transmission layer that is largely missed by routine surveillance. Those findings indicate that malaria elimination efforts in Amazonian forest settings face additional challenges from cryptic reservoirs at the human–wildlife interface. Integrating sensitive molecular diagnostics and wildlife-informed surveillance within a One Health framework will be essential to reduce the risk for persistent transmission and reemergence in remote Indigenous territories.

Appendix Additional information about zoonotic and anthroponotic *Plasmodium* spp. circulation between wild primates and Indigenous community, Peruvian Amazon, 2007–2020.
