# Supplementary material for: Zoonotic and Anthroponotic Plasmodium spp. Circulation between Wild Primates and Indigenous Community, Peruvian Amazon, 2007–2020
Source: Emerg Infect Dis. 2026 May;32(5):707–19. doi: 10.3201/eid3205.251695 (PMC13175102; doi:10.3201/eid3205.251695)
Supplement: Appendix — Additional information about zoonotic and anthroponotic Plasmodium spp. circulation between wild primates and Indigenous community, Peruvian Amazon, 2007–2020. [file 25-1695-Techapp-s1.pdf]

Article DOI: https://doi.org/10.3201/eid3205.251695

EID cannot ensure accessibility for supplementary materials supplied by authors. Readers who have difficulty accessing supplementary content should contact the authors for assistance.

Zoonotic and Anthroponotic *Plasmodium* spp. Circulation between Wild Primates and Indigenous Community, Peruvian Amazon, 2007–2020

Appendix

Supplementary Methods and Results

Detailed laboratory protocols for this study (available at https://www.protocols.io) include the following.

- DNA extraction from dried blood spots (DBS): https://dx.doi.org/10.17504/protocols.io.dm6gpbbr5lzp/v1
- Nested PCR for cytb gene amplification: https://dx.doi.org/10.17504/protocols.io.bp2l619k5vqe/v1
- Nested PCR for cox3 gene amplification: https://dx.doi.org/10.17504/protocols.io.4r3l2ooxpv1y/v1

Appendix Table 1. Primer sequences for amplification of the *cox3* gene of *Plasmodium* spp.

Protocol	Species	Primer sequence	bp
nPCR *cox3*	*Plasmodium* spp.	SPPF: 5’- CTC GCC ATT TGA TAG CGG TTA ACC-3’<br>SPPR: 5’- CCT GTT ATC CCC GGC GAA CCT TC-3’	940
	P. vivax/P. simium	F: 5'-CTA GCT TTT AAC ACA ATA TTA TTG TCT ATA C-3'<br>R: 5'-GTT CTT TTT CTA TTC AGA ATA ATG AAT ATA T-3'	87
	P. falciparum	F: 5'-GAA CAC AAT TGT CTA TTC GTA CAA TTA TTC-3'<br>R: 5'-CTT CTA CCG AAT GGT TTA TAA ATT CTT TC-3'	193
	P. brasilianum/P. malariae	F: 5'-CTA GCT TTG TAC ACA AAT TAA TTC GTC TAC-3'<br>R: 5'-CTT TAT AAG AAT GAT AGA TAT TTA TGA CAT A-3'	233

Primers used for nested PCR targeting cox3, adapted from Isozumi et al. (*1*), including genus-specific and species-specific sets.

Appendix Table 2. Metadata and GenBank accession numbers for *cytb* sequences of *Plasmodium* spp.

N	Sequence ID	Host	Isolate	Gene	GenBank Accession	Length (bp)	% High Quality	% Pairwise Identity	Annotation (CDS range)
1	Plasmodium sp. - Homo sapiens (human PC 9939)	*Homo sapiens*	9939	*cytochrome b*	PV769968	853	98.9%	99.9%	125..>853
2	Plasmodium sp. - Homo sapiens (human PC 9936)	*Homo sapiens*	9936	*cytochrome b*	PV769967	857	98.8%	100.0%	125..>857
3	Plasmodium sp. - Homo sapiens (human PC 9935)	*Homo sapiens*	9935	*cytochrome b*	PV769966	829	97.8%	99.2%	116..>829
4	Plasmodium sp. - Homo sapiens (human 9912)	*Homo sapiens*	9912	*cytochrome b*	PV769965	853	97.4%	99.9%	120..>853
5	Plasmodium sp. - Homo sapiens (human 7606)	*Homo sapiens*	7606	*cytochrome b*	PV769964	870	93.3%	98.8%	128..>870
6	Plasmodium sp. - Homo sapiens (human 7605)	*Homo sapiens*	7605	*cytochrome b*	PV769963	802	97.9%	100.0%	120..>802
7	Plasmodium sp. - Homo sapiens (human 7603)	*Homo sapiens*	7603	*cytochrome b*	PV769962	832	96.9%	99.5%	120..>832
8	Plasmodium sp. - Homo sapiens (human 7602)	*Homo sapiens*	7602	*cytochrome b*	PV769961	792	95.5%	99.7%	130..>792
9	Plasmodium sp. - Homo sapiens (human 7301)	*Homo sapiens*	7301	*cytochrome b*	PV769960	791	97.0%	98.8%	<154..>791
10	Plasmodium sp. - Homo sapiens (human 6803)	*Homo sapiens*	6803	*cytochrome b*	PV769959	863	98.0%	99.7%	123..>863
11	Plasmodium sp. - Homo sapiens (human 6402)	*Homo sapiens*	6402	*cytochrome b*	PV769958	511	57.1%	100.0%	<1..>511
12	Plasmodium sp. - Homo sapiens (human 6204)	*Homo sapiens*	6204	*cytochrome b*	PV769957	853	98.9%	99.2%	121..>853
13	Plasmodium sp. - Homo sapiens (human 6104)	*Homo sapiens*	6104	*cytochrome b*	PV769956	861	99.8%	98.6%	129..>861
14	Plasmodium sp. - Homo sapiens (human 6007)	*Homo sapiens*	6007	*cytochrome b*	PV769955	853	97.3%	100.0%	121..>853
15	Plasmodium sp. - Homo sapiens (human 6006)	*Homo sapiens*	6006	*cytochrome b*	PV769954	847	98.0%	99.2%	118..>847
16	Plasmodium sp. - Homo sapiens (human 6002)	*Homo sapiens*	6002	*cytochrome b*	PV769953	772	99.1%	98.9%	<131..>772
17	Plasmodium sp. - Homo sapiens (human 6001)	*Homo sapiens*	6001	*cytochrome b*	PV769952	852	97.7%	99.3%	125..>852
18	Plasmodium sp. - Homo sapiens (human 5906)	*Homo sapiens*	5906	*cytochrome b*	PV769951	848	99.3%	99.5%	117..>848
19	Plasmodium sp. - Homo sapiens (human 5905)	*Homo sapiens*	5905	*cytochrome b*	PV769950	850	99.4%	99.4%	118..>850
20	Plasmodium sp. - Homo sapiens (human 5904)	*Homo sapiens*	5904	*cytochrome b*	PV769949	822	99.9%	99.9%	<160..>822
21	Plasmodium sp. - Homo sapiens (human 5901)	*Homo sapiens*	5901	*cytochrome b*	PV769948	848	97.2%	99.6%	135..>848
22	Plasmodium sp. - Homo sapiens (human 5802)	*Homo sapiens*	5802	*cytochrome b*	PV769947	849	96.3%	99.5%	118..>849
23	Plasmodium sp. - Homo sapiens (human 5703)	*Homo sapiens*	5703	*cytochrome b*	PV769946	692	81.4%	99.0%	<1..>692

N	Sequence ID	Host	Isolate	Gene	GenBank Accession	Length (bp)	% High Quality	% Pairwise Identity	Annotation (CDS range)
24	Plasmodium sp. - Homo sapiens (human 5109)	*Homo sapiens*	5109	*cytochrome b*	PV769945	857	97.9%	99.9%	130..>857
25	Plasmodium sp. - Homo sapiens (human 5108)	*Homo sapiens*	5108	*cytochrome b*	PV769944	837	98.2%	99.5%	121..>837
26	Plasmodium sp. - Homo sapiens (human 5106)	*Homo sapiens*	5106	*cytochrome b*	PV769943	855	99.9%	99.8%	124..>855
27	Plasmodium sp. - Homo sapiens (human 5105)	*Homo sapiens*	5105	*cytochrome b*	PV769942	863	98.4%	99.7%	130..>863
28	Plasmodium sp. - Homo sapiens (human 5101)	*Homo sapiens*	5101	*cytochrome b*	PV769941	859	99.4%	99.9%	131..>859
29	Plasmodium sp. - Homo sapiens (human 4902)	*Homo sapiens*	4902	*cytochrome b*	PV769940	853	97.9%	99.4%	124..>853
30	Plasmodium sp. - Homo sapiens (human 4308)	*Homo sapiens*	4308	*cytochrome b*	PV769939	849	97.6%	99.7%	121..>849
31	Plasmodium sp. - Homo sapiens (human 4303)	*Homo sapiens*	4303	*cytochrome b*	PV769938	856	98.5%	98.9%	128..>856
32	Plasmodium sp. - Homo sapiens (human 4302)	*Homo sapiens*	4302	*cytochrome b*	PV769937	852	98.9%	99.1%	125..>852
33	Plasmodium sp. - Homo sapiens (human 3904)	*Homo sapiens*	3904	*cytochrome b*	PV769936	852	98.8%	99.8%	124..>852
34	Plasmodium sp. - Homo sapiens (human 3903)	*Homo sapiens*	3903	*cytochrome b*	PV769935	877	98.1%	99.4%	146..>877
35	Plasmodium sp. - Homo sapiens (human 3901)	*Homo sapiens*	3901	*cytochrome b*	PV769934	850	97.8%	99.5%	121..>850
36	Plasmodium sp. - Homo sapiens (human 3503)	*Homo sapiens*	3503	*cytochrome b*	PV769933	848	98.7%	99.8%	121..>848
37	Plasmodium sp. - Homo sapiens (human 3101)	*Homo sapiens*	3101	*cytochrome b*	PV769932	847	98.1%	99.7%	120..>847
38	Plasmodium sp. - Homo sapiens (human 2503)	*Homo sapiens*	2503	*cytochrome b*	PV769931	857	99.1%	99.9%	<124..>857
39	Plasmodium sp. - Homo sapiens (human 2201)	*Homo sapiens*	2201	*cytochrome b*	PV769930	857	99.5%	99.7%	122..>857
40	Plasmodium sp. - Homo sapiens (human 2002)	*Homo sapiens*	2002	*cytochrome b*	PV769929	834	98.7%	99.2%	125..>834
41	Plasmodium sp. - Homo sapiens (human 2001)	*Homo sapiens*	2001	*cytochrome b*	PV769928	832	98.3%	99.3%	120..>832
42	Plasmodium sp. - Homo sapiens (human 1913)	*Homo sapiens*	1913	*cytochrome b*	PV769927	859	97.3%	99.6%	117..>859
43	Plasmodium sp. - Homo sapiens (human 1912)	*Homo sapiens*	1912	*cytochrome b*	PV769926	834	99.8%	99.9%	122..>834
44	Plasmodium sp. - Homo sapiens (human 1909)	*Homo sapiens*	1909	*cytochrome b*	PV769925	849	98.8%	99.6%	120..>849
45	Plasmodium sp. - Homo sapiens (human 1905)	*Homo sapiens*	1905	*cytochrome b*	PV769924	861	100.0%	100.0%	130..>861
46	Plasmodium sp. - Homo sapiens (human 1901)	*Homo sapiens*	1901	*cytochrome b*	PV769923	860	98.0%	99.2%	131..>860

N	Sequence ID	Host	Isolate	Gene	GenBank Accession	Length (bp)	% High Quality	% Pairwise Identity	Annotation (CDS range)
47	Plasmodium sp. - Homo sapiens (human 1808)	*Homo sapiens*	1808	*cytochrome b*	PV769922	800	97.6%	99.4%	117..>800
48	Plasmodium sp. - Homo sapiens (human 1701)	*Homo sapiens*	1701	*cytochrome b*	PV769921	828	95.7%	98.7%	116..>828
49	Plasmodium sp. - Homo sapiens (human 1501)	*Homo sapiens*	1501	*cytochrome b*	PV769920	856	99.3%	99.2%	125..>856
50	Plasmodium sp. - Homo sapiens (human 1005)	*Homo sapiens*	1005	*cytochrome b*	PV769919	856	99.3%	99.8%	125..>856
51	Plasmodium sp. - Homo sapiens (human 1001)	*Homo sapiens*	1001	*cytochrome b*	PV769918	857	99.6%	99.7%	125..>857
52	Plasmodium sp. - Homo sapiens (human 0906)	*Homo sapiens*	906	*cytochrome b*	PV769917	842	98.2%	99.8%	117..>842
53	Plasmodium sp. - Homo sapiens (human 0903F3)	*Homo sapiens*	903	*cytochrome b*	PV769916	814	94.8%	NA	118..>814
54	Plasmodium sp. - Homo sapiens (human 0801)	*Homo sapiens*	801	*cytochrome b*	PV769915	861	98.7%	99.7%	130..>861
55	Plasmodium sp. - Homo sapiens (human 0703)	*Homo sapiens*	703	*cytochrome b*	PV769914	830	99.3%	99.6%	114..>830
56	Plasmodium sp. - Homo sapiens (human 0701)	*Homo sapiens*	701	*cytochrome b*	PV769913	859	97.6%	99.4%	127..>859
57	Plasmodium sp. - Homo sapiens (human 0612)	*Homo sapiens*	612	*cytochrome b*	PV769912	843	99.5%	99.6%	114..>843
58	Plasmodium sp. - Homo sapiens (human 0607)	*Homo sapiens*	607	*cytochrome b*	PV769911	849	99.6%	99.6%	121..>849
59	Plasmodium sp. - Homo sapiens (human 0605)	*Homo sapiens*	605	*cytochrome b*	PV769910	852	97.8%	98.8%	120..>852
60	Plasmodium sp. - Homo sapiens (human 0602)	*Homo sapiens*	602	*cytochrome b*	PV769909	856	99.2%	99.6%	125..>856
61	Plasmodium sp. - Homo sapiens (human 0601)	*Homo sapiens*	601	*cytochrome b*	PV769908	854	99.5%	99.8%	125..>854
62	Plasmodium sp. - Homo sapiens (human 0505)	*Homo sapiens*	505	*cytochrome b*	PV769907	851	98.2%	99.8%	120..>851
63	Plasmodium sp. - Homo sapiens (human 0503)	*Homo sapiens*	503	*cytochrome b*	PV769906	837	98.4%	99.7%	122..>837
64	Plasmodium sp. - A. chamek (maquisapa_303_R1)	*Ateles Chamek*	maquisapa_303_R1	*cytochrome b*	PV786592	790	94.7%	NA	132..>790
65	Plasmodium sp. - A. chamek (maquisapa_548)	*Ateles Chamek*	maquisapa_548	*cytochrome b*	PV786591	828	98.3%	99.6%	105..>828
66	Plasmodium sp. - A. chamek (maquisapa_1049)	*Ateles Chamek*	maquisapa_1049	*cytochrome b*	PV786590	849	96.2%	99.6%	120..>849
67	Plasmodium sp. - A. chamek (maquisapa_1050)	*Ateles Chamek*	maquisapa_1050	*cytochrome b*	PV786589	841	99.9%	99.9%	109..>841
68	Plasmodium sp. - A. chamek (maquisapa_1051_R1)	*Ateles Chamek*	maquisapa_1051_R1	*cytochrome b*	PV786588	811	94.1%	NA	135..>811
69	Plasmodium sp. - A. chamek (maquisapa_1052)	*Ateles Chamek*	maquisapa_1052	*cytochrome b*	PV786587	881	94.3%	95.7%	142..>881

N	Sequence ID	Host	Isolate	Gene	GenBank Accession	Length (bp)	% High Quality	% Pairwise Identity	Annotation (CDS range)
70	Plasmodium sp. - A. chamek (maquisapa_1058_R1)	*Ateles Chamek*	maquisapa_1058_R1	*cytochrome b*	PV786586	353	52.4%	NA	<118..>353
71	Plasmodium sp. - A. chamek (maquisapa_1117)	*Ateles Chamek*	maquisapa_1117	*cytochrome b*	PV786585	823	98.7%	99.6%	91..>823
72	Plasmodium sp. - A. chamek (maquisapa_1157_R1)	*Ateles Chamek*	maquisapa_1157_R1	*cytochrome b*	PV786584	815	97.7%	NA	118..>815
73	Plasmodium sp. - A. chamek (maquisapa_032014)	*Ateles Chamek*	maquisapa_032014	*cytochrome b*	PV786583	849	98.8%	99.9%	117..>849
74	Plasmodium sp. - A. chamek (maquisapa_042014)	*Ateles Chamek*	maquisapa_042014	*cytochrome b*	PV786582	858	99.7%	99.9%	129..>858
75	Plasmodium sp. - A. chamek (maquisapa_192014)	*Ateles Chamek*	maquisapa_192014	*cytochrome b*	PV786581	824	97.8%	99.2%	110..>824
76	Plasmodium sp. - A. chamek (maquisapa_A115)	*Ateles Chamek*	maquisapa_A115	*cytochrome b*	PV786580	817	98.5%	99.9%	101..>817
77	Plasmodium sp. - A. chamek (maquisapa_N026)	*Ateles Chamek*	maquisapa_N026	*cytochrome b*	PV786579	821	97.3%	99.9%	111..>821
78	Plasmodium sp. - A. seniculus (coto_288)	*Alouatta seniculus*	coto_288	*cytochrome b*	PV786578	841	96.6%	100.0%	114..>841
79	Plasmodium sp. - A. seniculus (coto_482_R1)	*Alouatta seniculus*	coto_482_R1	*cytochrome b*	PV786577	829	94.9%	NA	127..>829
80	Plasmodium sp. - A. seniculus (coto_483)	*Alouatta seniculus*	coto_483	*cytochrome b*	PV786576	861	94.9%	99.5%	130..>861
81	Plasmodium sp. - A. seniculus (coto_484_R1)	*Alouatta seniculus*	coto_484_R1	*cytochrome b*	PV786575	813	98.2%	NA	118..>813
82	Plasmodium sp. - A. seniculus (coto_1008)	*Alouatta seniculus*	coto_1008	*cytochrome b*	PV786574	823	98.7%	100.0%	109..>823
83	Plasmodium sp. - A. seniculus (coto_1021_R1)	*Alouatta seniculus*	coto_1021_R1	*cytochrome b*	PV786573	791	96.2%	NA	<271..>791
84	Plasmodium sp. - A. seniculus (coto_1043)	*Alouatta seniculus*	coto_1043	*cytochrome b*	PV786572	827	96.0%	95.5%	121..>827
85	Plasmodium sp. - A. seniculus (coto_1077)	*Alouatta seniculus*	coto_1077	*cytochrome b*	PV786571	852	98.8%	100.0%	120..>852
86	Plasmodium sp. - A. seniculus (coto_1080)	*Alouatta seniculus*	coto_1080	*cytochrome b*	PV786570	852	98.4%	99.4%	120..>852
87	Plasmodium sp. - A. seniculus (coto_1121)	*Alouatta seniculus*	coto_1121	*cytochrome b*	PV786569	859	95.8%	98.9%	128..>859
88	Plasmodium sp. - A. seniculus (coto_102014)	*Alouatta seniculus*	coto_102014	*cytochrome b*	PV786568	837	99.0%	99.7%	110..>837
89	Plasmodium sp. - A. seniculus (coto_A67_F1)	*Alouatta seniculus*	coto_A67_F1	*cytochrome b*	PV786567	773	82.1%	NA	<109..>773
90	Plasmodium sp. - A. seniculus (coto_EN1)	*Alouatta seniculus*	coto_EN1	*cytochrome b*	PV786566	856	97.8%	99.9%	127..>856
91	Plasmodium sp. - A. seniculus (coto_SN)	*Alouatta seniculus*	coto_SN	*cytochrome b*	PV786565	832	96.2%	98.5%	117..>832
92	Plasmodium sp. - C. albifrons (machínb_505)	*Cebus albifrons*	machínb_505	*cytochrome b*	PV786564	800	98.0%	NA	117..>800

N	Sequence ID	Host	Isolate	Gene	GenBank Accession	Length (bp)	% High Quality	% Pairwise Identity	Annotation (CDS range)
93	Plasmodium sp. - C. albifrons (machínb_603)	*Cebus albifrons*	machínb_603	*cytochrome b*	PV786563	814	93.6%	NA	112..>814
94	Plasmodium sp. - C. albifrons (machínblanco_1010)	*Cebus albifrons*	machínblanco_1010	*cytochrome b*	PV786562	855	96.8%	99.7%	111..>855
95	Plasmodium sp. - C. albifrons (machínblanco_1022)	*Cebus albifrons*	machínblanco_1022	*cytochrome b*	PV786561	848	98.2%	99.7%	117..>848
96	Plasmodium sp. - C. albifrons (machínblanco_1030)	*Cebus albifrons*	machínblanco_1030	*cytochrome b*	PV786560	831	97.7%	99.9%	117..>831
97	Plasmodium sp. - C. albifrons (machínblanco_1031)	*Cebus albifrons*	machínblanco_1031	*cytochrome b*	PV786559	873	97.7%	99.2%	141..>873
98	Plasmodium sp. - C. albifrons (machínblanco_1086)	*Cebus albifrons*	machínblanco_1086	*cytochrome b*	PV786558	828	93.8%	99.9%	120..>828
99	Plasmodium sp. - C. albifrons (machínblanco_1122)	*Cebus albifrons*	machínblanco_1122	*cytochrome b*	PV786557	850	97.8%	98.6%	121..>850
100	Plasmodium sp. - C. albifrons (machínblanco_1132)	*Cebus albifrons*	machínblanco_1132	*cytochrome b*	PV786556	860	87.6%	100.0%	128..>860
101	Plasmodium sp. - C. albifrons (machínblanco_EN1b)	*Cebus albifrons*	machínblanco_EN1b	*cytochrome b*	PV786555	847	97.5%	98.2%	117..>847
102	Plasmodium sp. - C. albifrons (machínblanco_N038)	*Cebus albifrons*	machínblanco_N038	*cytochrome b*	PV786554	864	96.9%	99.9%	132..>864
103	Plasmodium sp. - C. albifrons (machínblanco_N039)	*Cebus albifrons*	machínblanco_N039	*cytochrome b*	PV786553	845	98.0%	99.9%	113..>845
104	Plasmodium sp. - C. calvus (uacari_008)	*Cacajao calvus ucayalii*	uacari_008	*cytochrome b*	PV786552	831	99.5%	99.7%	121..>831
105	Plasmodium sp. - C. calvus (uacari_011)	*Cacajao calvus ucayalii*	uacari_011	*cytochrome b*	PV786551	838	99.5%	99.7%	110..>838
106	Plasmodium sp. - C. calvus (uacari_023)	*Cacajao calvus ucayalii*	uacari_023	*cytochrome b*	PV786550	855	99.5%	99.8%	123..>855
107	Plasmodium sp. - C. calvus (uacari_110)	*Cacajao calvus ucayalii*	uacari_110	*cytochrome b*	PV786549	841	98.9%	99.5%	112..>841
108	Plasmodium sp. - C. calvus (uacari_123)	*Cacajao calvus ucayalii*	uacari_123	*cytochrome b*	PV786548	859	99.0%	99.8%	127..>859
109	Plasmodium sp. - C. calvus (uacari_480)	*Cacajao calvus ucayalii*	uacari_480	*cytochrome b*	PV786547	849	99.6%	98.7%	117..>849
110	Plasmodium sp. - C. calvus (uacari_488)	*Cacajao calvus ucayalii*	uacari_488	*cytochrome b*	PV786546	843	98.9%	99.3%	110..>843
111	Plasmodium sp. - C. calvus (uacari_581)	*Cacajao calvus ucayalii*	uacari_581	*cytochrome b*	PV786545	842	98.6%	98.9%	110..>842
112	Plasmodium sp. - C. calvus (uacari_582)	*Cacajao calvus ucayalii*	uacari_582	*cytochrome b*	PV786544	746	96.5%	97.5%	33..>746
113	Plasmodium sp. - C. calvus (uacari_583)	*Cacajao calvus ucayalii*	uacari_583	*cytochrome b*	PV786543	838	97.7%	99.5%	111..>838
114	Plasmodium sp. - C. calvus (uacari_586)	*Cacajao calvus ucayalii*	uacari_586	*cytochrome b*	PV786542	826	98.7%	99.6%	117..>826
115	Plasmodium sp. - C. calvus (uacari_587)	*Cacajao calvus ucayalii*	uacari_587	*cytochrome b*	PV786541	771	97.9%	99.1%	116..>771

N	Sequence ID	Host	Isolate	Gene	GenBank Accession	Length (bp)	% High Quality	% Pairwise Identity	Annotation (CDS range)
116	Plasmodium sp. - C. calvus (uacari_589)	*Cacajao calvus ucayalii*	uacari_589	*cytochrome b*	PV786540	826	99.3%	99.9%	112..>826
117	Plasmodium sp. - C. calvus (uacari_1011)	*Cacajao calvus ucayalii*	uacari_1011	*cytochrome b*	PV786539	834	97.4%	99.9%	111..>834
118	Plasmodium sp. - C. calvus (uacari_1018)	*Cacajao calvus ucayalii*	uacari_1018	*cytochrome b*	PV786538	820	97.3%	99.5%	106..>820
119	Plasmodium sp. - C. calvus (uacari_1087)	*Cacajao calvus ucayalii*	uacari_1087	*cytochrome b*	PV786537	859	99.2%	100.0%	126..>859
120	Plasmodium sp. - C. calvus (uacari_1105)	*Cacajao calvus ucayalii*	uacari_1105	*cytochrome b*	PV786536	832	97.2%	99.9%	105..>832
121	Plasmodium sp. - C. calvus (uacari_N109_R1)	*Cacajao calvus ucayalii*	uacari_N109_R1	*cytochrome b*	PV786535	785	75.8%	NA	83..>785
122	Plasmodium sp. - C. calvus (uacari_N110)	*Cacajao calvus ucayalii*	uacari_N110	*cytochrome b*	PV786534	813	96.4%	100.0%	113..>813
123	Plasmodium sp. - C. calvus (uacari_N111)	*Cacajao calvus ucayalii*	uacari_N111	*cytochrome b*	PV786533	864	96.6%	99.7%	132..>864
124	Plasmodium sp. - C. cupreus (tocón _1046)	*Plecturocebus cupreus*	tocón_1046	*cytochrome b*	PV786532	859	95.5%	99.7%	127..>859
125	Plasmodium sp. - C. cupreus (tocón_1048_F1)	*Plecturocebus cupreus*	tocón_1048_F1	*cytochrome b*	PV786531	806	87.5%	NA	88..>806
126	Plasmodium sp. - C. cupreus (tocón_1075)	*Plecturocebus cupreus*	tocón_1075	*cytochrome b*	PV786530	854	96.6%	100.0%	125..>854
127	Plasmodium sp. - L. fuscicolis (pichico_052014_R1)	*Leontocebus fuscicolis*	pichico_052014_R1	*cytochrome b*	PV786529	731	89.9%	NA	<176..>731
128	Plasmodium sp. - L. poeppigii (monochoro_026)	*Lagothrix lagothrica poeppigi*	monochoro_026	*cytochrome b*	PV786528	859	97.7%	96.0%	127..>859
129	Plasmodium sp. - L. poeppigii (monochoro_064)	*Lagothrix lagothrica poeppigi*	monochoro_064	*cytochrome b*	PV786527	854	98.4%	99.9%	126..>854
130	Plasmodium sp. - L. poeppigii (monochoro_069)	*Lagothrix lagothrica poeppigi*	monochoro_069	*cytochrome b*	PV786526	851	98.0%	98.8%	140..>851
131	Plasmodium sp. - L. poeppigii (monochoro_083)	*Lagothrix lagothrica poeppigi*	monochoro_083	*cytochrome b*	PV786525	858	98.7%	99.9%	129..>858
132	Plasmodium sp. - L. poeppigii (monochoro_122)	*Lagothrix lagothrica poeppigi*	monochoro_122	*cytochrome b*	PV786524	851	99.1%	99.8%	118..>851
133	Plasmodium sp. - L. poeppigii (monochoro_144)	*Lagothrix lagothrica poeppigi*	monochoro_144	*cytochrome b*	PV786523	852	97.5%	99.8%	125..>852
134	Plasmodium sp. - L. poeppigii (monochoro_151)	*Lagothrix lagothrica poeppigi*	monochoro_151	*cytochrome b*	PV786522	846	99.3%	99.9%	114..>846
135	Plasmodium sp. - L. poeppigii (monochoro_153)	*Lagothrix lagothrica poeppigi*	monochoro_153	*cytochrome b*	PV786521	845	98.3%	99.9%	113..>845
136	Plasmodium sp. - L. poeppigii (monochoro_407_R1)	*Lagothrix lagothrica poeppigi*	monochoro_407_R1	*cytochrome b*	PV786520	751	89.9%	NA	121..>751
137	Plasmodium sp. - L. poeppigii (monochoro_481)	*Lagothrix lagothrica poeppigi*	monochoro_481	*cytochrome b*	PV786519	840	98.1%	99.2%	112..>840
138	Plasmodium sp. - L. poeppigii (monochoro_496)	*Lagothrix lagothrica poeppigi*	monochoro_496	*cytochrome b*	PV786518	841	98.2%	99.7%	112..>841

N	Sequence ID	Host	Isolate	Gene	GenBank Accession	Length (bp)	% High Quality	% Pairwise Identity	Annotation (CDS range)
139	Plasmodium sp. - L. poeppigii (monochoro_507)	*Lagothrix lagothrica poeppigi*	monochoro_507	*cytochrome b*	PV786517	854	97.7%	99.9%	127..>854
140	Plasmodium sp. - L. poeppigii (monochoro_519)	*Lagothrix lagothrica poeppigi*	monochoro_519	*cytochrome b*	PV786516	852	95.9%	100.0%	125..>852
141	Plasmodium sp. - L. poeppigii (monochoro_527)	*Lagothrix lagothrica poeppigi*	monochoro_527	*cytochrome b*	PV786515	844	99.5%	100.0%	112..>844
142	Plasmodium sp. - L. poeppigii (monochoro_535)	*Lagothrix lagothrica poeppigi*	monochoro_535	*cytochrome b*	PV786514	845	96.0%	99.7%	118..>845
143	Plasmodium sp. - L. poeppigii (monochoro_536)	*Lagothrix lagothrica poeppigi*	monochoro_536	*cytochrome b*	PV786513	854	98.8%	99.7%	121..>854
144	Plasmodium sp. - L. poeppigii (monochoro_541_R1)	*Lagothrix lagothrica poeppigi*	(monochoro_541_R1	*cytochrome b*	PV786512	786	91.9%	NA	128..>786
145	Plasmodium sp. - L. poeppigii (monochoro_552)	*Lagothrix lagothrica poeppigi*	monochoro_552	*cytochrome b*	PV786511	842	97.9%	100.0%	110..>842
146	Plasmodium sp. - L. poeppigii (monochoro_1006)	*Lagothrix lagothrica poeppigi*	monochoro_1006	*cytochrome b*	PV786510	849	97.1%	100.0%	120..>849
147	Plasmodium sp. - L. poeppigii (monochoro_1007)	*Lagothrix lagothrica poeppigi*	monochoro_1007	*cytochrome b*	PV786509	862	97.8%	99.9%	130..>862
148	Plasmodium sp. - L. poeppigii (monochoro_1034)	*Lagothrix lagothrica poeppigi*	monochoro_1034	*cytochrome b*	PV786508	807	97.5%	99.2%	116..>807
149	Plasmodium sp. - L. poeppigii (monochoro_1037)	*Lagothrix lagothrica poeppigi*	monochoro_1037	*cytochrome b*	PV786507	845	99.4%	99.9%	130..>845
150	Plasmodium sp. - L. poeppigii (monochoro_1038)	*Lagothrix lagothrica poeppigi*	monochoro_1038	*cytochrome b*	PV786506	865	97.1%	100.0%	133..>865
151	Plasmodium sp. - L. poeppigii (monochoro_1056)	*Lagothrix lagothrica poeppigi*	monochoro_1056	*cytochrome b*	PV786505	848	97.3%	99.5%	116..>848
152	Plasmodium sp. - L. poeppigii (monochoro_1060)	*Lagothrix lagothrica poeppigi*	monochoro_1060	*cytochrome b*	PV786504	801	96.4%	98.1%	88..>801
153	Plasmodium sp. - L. poeppigii (monochoro_1066_F1)	*Lagothrix lagothrica poeppigi*	monochoro_1066_F1	*cytochrome b*	PV786503	813	96.6%	NA	81..>813
154	Plasmodium sp. - L. poeppigii (monochoro_1079)	*Lagothrix lagothrica poeppigi*	monochoro_1079	*cytochrome b*	PV786502	849	92.7%	98.0%	116..>849
155	Plasmodium sp. - L. poeppigii (monochoro_1101)	*Lagothrix lagothrica poeppigi*	monochoro_1101	*cytochrome b*	PV786501	838	97.1%	99.6%	109..>838
156	Plasmodium sp. - L. poeppigii (monochoro_1107)	*Lagothrix lagothrica poeppigi*	monochoro_1107	*cytochrome b*	PV786500	844	99.4%	99.8%	112..>844
157	Plasmodium sp. - L. poeppigii (monochoro_1125)	*Lagothrix lagothrica poeppigi*	monochoro_1125	*cytochrome b*	PV786499	864	98.3%	99.8%	132..>864
158	Plasmodium sp. - L. poeppigii (monochoro_1126)	*Lagothrix lagothrica poeppigi*	monochoro_1126	*cytochrome b*	PV786498	854	98.7%	99.7%	125..>854
159	Plasmodium sp. - L. poeppigii (monochoro_1130)	*Lagothrix lagothrica poeppigi*	monochoro_1130	*cytochrome b*	PV786497	860	98.8%	99.9%	128..>860
160	Plasmodium sp. - L. poeppigii (monochoro_1133_F1)	*Lagothrix lagothrica poeppigi*	monochoro_1133_F1	*cytochrome b*	PV786496	829	90.8%	NA	97..>829
161	Plasmodium sp. - L. poeppigii (monochoro_A100)	*Lagothrix lagothrica poeppigi*	monochoro_A100	*cytochrome b*	PV786495	859	99.0%	99.7%	130..>859

N	Sequence ID	Host	Isolate	Gene	GenBank Accession	Length (bp)	% High Quality	% Pairwise Identity	Annotation (CDS range)
162	Plasmodium sp. - L. poeppigii (monochoro_N047_R1)	*Lagothrix lagothrica poeppigi*	monochoro_N047_R1	*cytochrome b*	PV786494	809	97.7%	NA	118..>809
163	Plasmodium sp. - L. poeppigii (monochoro_N057)	*Lagothrix lagothrica poeppigi*	monochoro_N057	*cytochrome b*	PV786493	838	93.2%	99.9%	103..>838
164	Plasmodium sp. - L. poeppigii (monochoro_N060_R1)	*Lagothrix lagothrica poeppigi*	monochoro_N060_R1	*cytochrome b*	PV786492	807	98.6%	NA	117..>807
165	Plasmodium sp. - L. poeppigii (monochoro_N061)	*Lagothrix lagothrica poeppigi*	monochoro_N061	*cytochrome b*	PV786491	830	98.0%	99.9%	102..>830
166	Plasmodium sp. - L. poeppigii (monochoro_N063)	*Lagothrix lagothrica poeppigi*	monochoro_N063	*cytochrome b*	PV786490	841	98.5%	99.9%	109..>841
167	Plasmodium sp. - L. poeppigii (monochoro_N066)	*Lagothrix lagothrica poeppigi*	monochoro_N066	*cytochrome b*	PV786489	852	95.9%	99.7%	118..>852
168	Plasmodium sp. - L. poeppigii (monochoro_N067)	*Lagothrix lagothrica poeppigi*	monochoro_N067	*cytochrome b*	PV786488	844	98.3%	99.9%	113..>844
169	Plasmodium sp. - L. poeppigii (monochoro_N068)	*Lagothrix lagothrica poeppigi*	monochoro_N068	*cytochrome b*	PV786487	850	97.8%	99.7%	118..>850
170	Plasmodium sp. - L. poeppigii (monochoro_N073_R1)	*Lagothrix lagothrica poeppigi*	monochoro_N073_R1	*cytochrome b*	PV786486	808	97.3%	NA	118..>808
171	Plasmodium sp. - L. poeppigii (monochoro_N086)	*Lagothrix lagothrica poeppigi*	monochoro_N086	*cytochrome b*	PV786485	800	99.4%	100.0%	110..>800
172	Plasmodium sp. - L. poeppigii (monochoro_N096)	*Lagothrix lagothrica poeppigi*	monochoro_N096	*cytochrome b*	PV786484	852	97.2%	99.9%	121..>852
173	Plasmodium sp. - L. poeppigii (monochoro_N101)	*Lagothrix lagothrica poeppigi*	monochoro_N101	*cytochrome b*	PV786483	854	97.8%	99.7%	123..>854
174	Plasmodium sp. - L. poeppigii (monochoro_N104)	*Lagothrix lagothrica poeppigi*	monochoro_N104	*cytochrome b*	PV786482	827	99.2%	99.7%	105..>827
175	Plasmodium sp. - P. monachus (huaponegro_238)	*Pithecia monachus*	huaponegro_238	*cytochrome b*	PV786481	826	99.0%	99.9%	118..>826
176	Plasmodium sp. - P. monachus (huaponegro_485)	*Pithecia monachus*	huaponegro_485	*cytochrome b*	PV786480	821	97.9%	99.0%	118..>821
177	Plasmodium sp. - P. monachus (huaponegro_486)	*Pithecia monachus*	huaponegro_486	*cytochrome b*	PV786479	847	97.8%	99.7%	118..>847
178	Plasmodium sp. - P. monachus (huaponegro_554)	*Pithecia monachus*	huaponegro_554	*cytochrome b*	PV786478	838	98.3%	99.2%	109..>838
179	Plasmodium sp. - P. monachus (huaponegro_1014)	*Pithecia monachus*	huaponegro_1014	*cytochrome b*	PV786477	820	99.3%	100.0%	<167..>820
180	Plasmodium sp. - P. monachus (huaponegro_1019)	*Pithecia monachus*	huaponegro_1019	*cytochrome b*	PV786476	840	98.3%	99.7%	112..>840
181	Plasmodium sp. - P. monachus (huaponegro_1089)	*Pithecia monachus*	huaponegro_1089	*cytochrome b*	PV786475	852	96.5%	99.9%	120..>852
182	Plasmodium sp. - P. monachus (huaponegro_1129)	*Pithecia monachus*	huaponegro_1129	*cytochrome b*	PV786474	844	97.5%	99.1%	117..>844
183	Plasmodium sp. - P. monachus (huaponegro_092014)	*Pithecia monachus*	huaponegro_092014	*cytochrome b*	PV786473	837	97.0%	99.6%	105..>837
184	Plasmodium sp. - S. macrocephalus (machínn_500)	*Sapajus macrocephalus*	machínnegro_500	*cytochrome b*	PV786472	835	99.6%	99.3%	121..>835

N	Sequence ID	Host	Isolate	Gene	GenBank Accession	Length (bp)	% High Quality	% Pairwise Identity	Annotation (CDS range)
185	Plasmodium sp. - S. macrocephalus (machínn_1015)	*Sapajus macrocephalus*	machínnegro_1015	*cytochrome b*	PV786471	841	98.2%	99.9%	125..>841
186	Plasmodium sp. - S. macrocephalus (machínn_1026)	*Sapajus macrocephalus*	machínn_1026	*cytochrome b*	PV786470	811	85.2%	NA	128..>811
187	Plasmodium sp. - S. macrocephalus (machínn_1028)	*Sapajus macrocephalus*	machínnegro_1028	*cytochrome b*	PV786469	848	96.2%	99.5%	116..>848
188	Plasmodium sp. - S. macrocephalus (machínn_1036)	*Sapajus macrocephalus*	machínnegro_1036	*cytochrome b*	PV786468	870	87.0%	99.2%	138..>870
189	Plasmodium sp. - S. macrocephalus (machínn_1039)	*Sapajus macrocephalus*	machínnegro_1039	*cytochrome b*	PV786467	854	98.4%	99.9%	125..>854
190	Plasmodium sp. - S. macrocephalus (machínn_1057)	*Sapajus macrocephalus*	machínnegro_1057	*cytochrome b*	PV786466	857	96.4%	99.7%	126..>857
191	Plasmodium sp. - S. macrocephalus (machínn_1078)	*Sapajus macrocephalus*	machínnegro_1078	*cytochrome b*	PV786465	848	96.7%	99.8%	116..>848
192	Plasmodium sp. - S. macrocephalus (machínn_1088)	*Sapajus macrocephalus*	machínnegro_1088	*cytochrome b*	PV786464	848	98·7%	99.9%	120..>848
193	Plasmodium sp. - S. macrocephalus (machínn_1097)	*Sapajus macrocephalus*	machínnegro_1097	*cytochrome b*	PV786463	835	97·5%	99.9%	120..>835
194	Plasmodium sp. - S. macrocephalus (machínn_1102)	*Sapajus macrocephalus*	machínn_1102	*cytochrome b*	PV786462	810	95.8%	NA	127..>810
195	Plasmodium sp. - S. macrocephalus (machínn_1103)	*Sapajus macrocephalus*	machínn_1103	*cytochrome b*	PV786461	795	84.9%	NA	148..>795
196	Plasmodium sp. - S. macrocephalus (machínn_1113)	*Sapajus macrocephalus*	machínn_1113	*cytochrome b*	PV786460	761	93.8%	NA	<106..>761
197	Plasmodium sp. - S. macrocephalus (machínn_1114)	*Sapajus macrocephalus*	machínnegro_1114	*cytochrome b*	PV786459	852	99.8%	100.0%	120..>852
198	Plasmodium sp. - S. macrocephalus (machínn_1127)	*Sapajus macrocephalus*	machínn_1127	*cytochrome b*	PV786458	787	96.1%	NA	84..>787
199	Plasmodium sp. - S. macrocephalus (machínn_1128)	*Sapajus macrocephalus*	machínnegro_1128	*cytochrome b*	PV786457	861	98.1%	100.0%	129..>861
200	Plasmodium sp. - S. macrocephalus (machínn_1134)	*Sapajus macrocephalus*	machínnegro_1134	*cytochrome b*	PV786456	854	98.5%	99.9%	126..>854
201	Plasmodium sp. - S. macrocephalus (machínn_1178)	*Sapajus macrocephalus*	machínnegro_1178	*cytochrome b*	PV786455	809	96.5%	100.0%	118..>809
202	Plasmodium sp. - S. macrocephalus (machínn_C121)	*Sapajus macrocephalus*	machínnegro_C121	*cytochrome b*	PV786454	850	98.8%	99.9%	118..>850
203	Plasmodium sp. - S. macrocephalus (machínn_N005)	*Sapajus macrocephalus*	machínn_N005	*cytochrome b*	PV786453	812	97.3%	NA	121..>812
204	Plasmodium sp. - S. macrodon (monofraile_473)	*Saimiri macrodon*	monofraile_473	*cytochrome b*	PV786452	836	98.7%	99.9%	122..>836
205	Plasmodium sp. - S. macrodon (monofraile_1025)	*Saimiri macrodon*	monofraile_1025	*cytochrome b*	PV786451	846	98.2%	100.0%	114..>846
206	Plasmodium sp. - S. macrodon (monofraile_1042)	*Saimiri macrodon*	monofraile_1042	*cytochrome b*	PV786450	860	89.8%	99.8%	128..>860
207	Plasmodium sp. - S. macrodon (monofraile_1062)	*Saimiri macrodon*	monofraile_1062	*cytochrome b*	PV786449	853	98.8%	100.0%	121..>853

N	Sequence ID	Host	Isolate	Gene	GenBank Accession	Length (bp)	% High Quality	% Pairwise Identity	Annotation (CDS range)
208	Plasmodium sp. - S. macrodon (monofraile_1063)	*Saimiri macrodon*	monofraile_1063	*cytochrome b*	PV786448	859	98.5%	100.0%	128..>859
209	Plasmodium sp. - S. macrodon (monofraile_CH13)	*Saimiri macrodon*	monofraile_CH13	*cytochrome b*	PV786447	856	96.6%	99.9%	124..>856

Sequences from humans and non-human primates (NHPs) from the Peruvian Amazon are generated by single-genome amplification (SGA).

Appendix Table 3. Temporal variation in *Plasmodium* prevalence among NHP hosts (2007–2020)

Host	*Plasmodium* species	CV
Cacajao calvus ucayalii	*P. brasilianum/P. malariae*	0.203
Lagothrix lagothrica poeppigii	*P. brasilianum/P. malariae*	0.257
Pithecia monachus	*P. brasilianum/P. malariae*	0.284
Plecturocebus cupreus	*P. vivax/P. simium*	0.346
Cebus albifrons	*P. vivax/P. simium*	0.365
Ateles chamek	*P. vivax/P. simium*	0.422
Alouatta seniculus	*P. vivax/P. simium*	0.508
Ateles chamek	*P. brasilianum/P. malariae*	0.519
Sapajus macrocephalus	*P. vivax/P. simium*	0.539
Cebus albifrons	*P. brasilianum/P. malariae*	0.549
Pithecia monachus	*P. vivax/P. simium*	0.606
Cacajao calvus ucayalii	*P. vivax/P. simium*	0.620
Sapajus macrocephalus	*P. brasilianum/P. malariae*	0.672
Alouatta seniculus	*P. brasilianum/P. malariae*	0.748
Saimiri macrodon	*P. brasilianum/P. malariae*	0.849
Lagothrix lagothrica poeppigi	*P. vivax/P. simium*	0.969

Coefficient of variation (CV) of annual prevalence estimates for each host-parasite pair, calculated across sampling years. Lower CV = more stable infection.

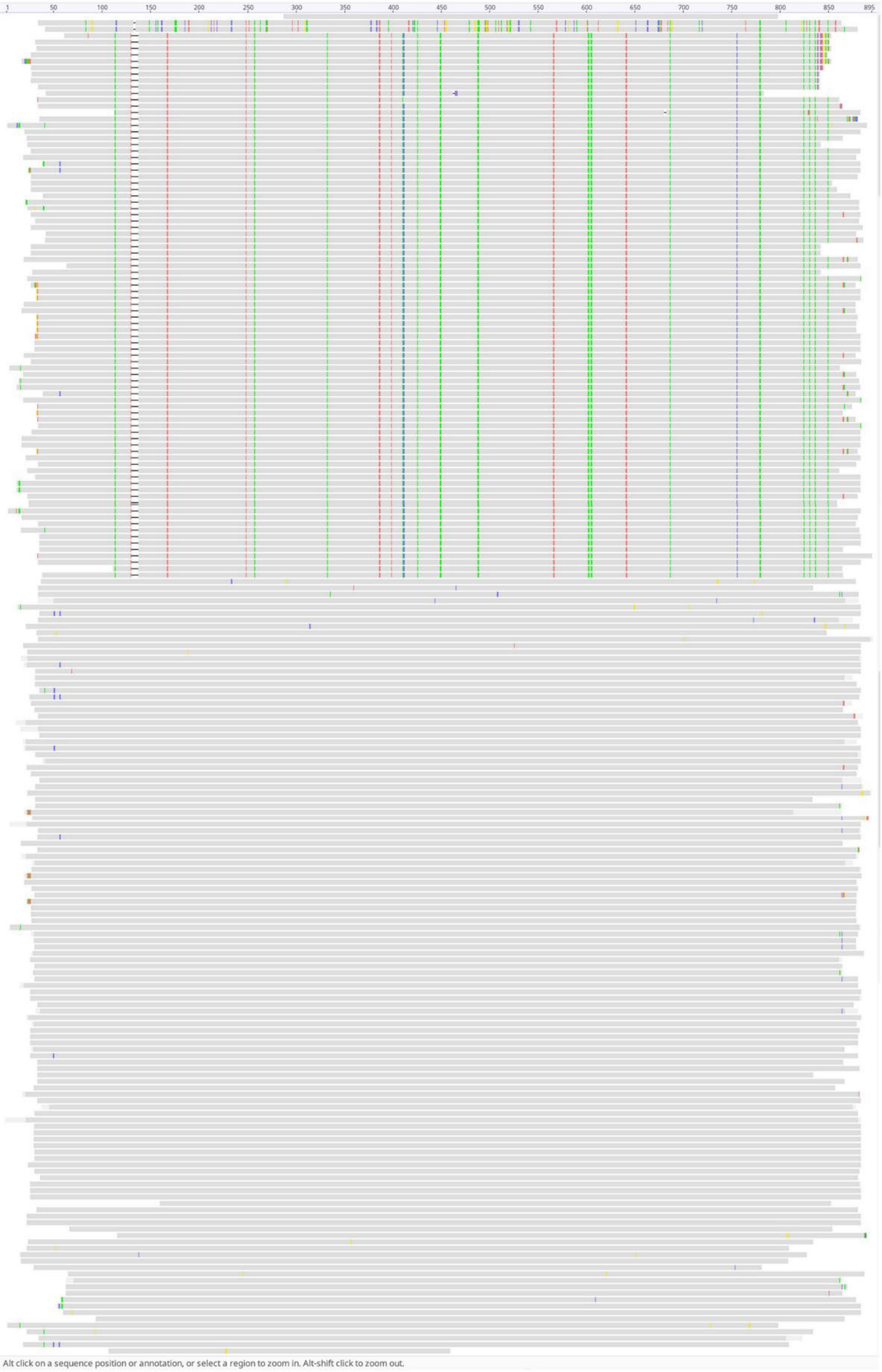

Appendix Figure 1. Primers used for nested PCR targeting cox3, adapted from Isozumi et al. (*1*), including genus-specific and species-specific sets.

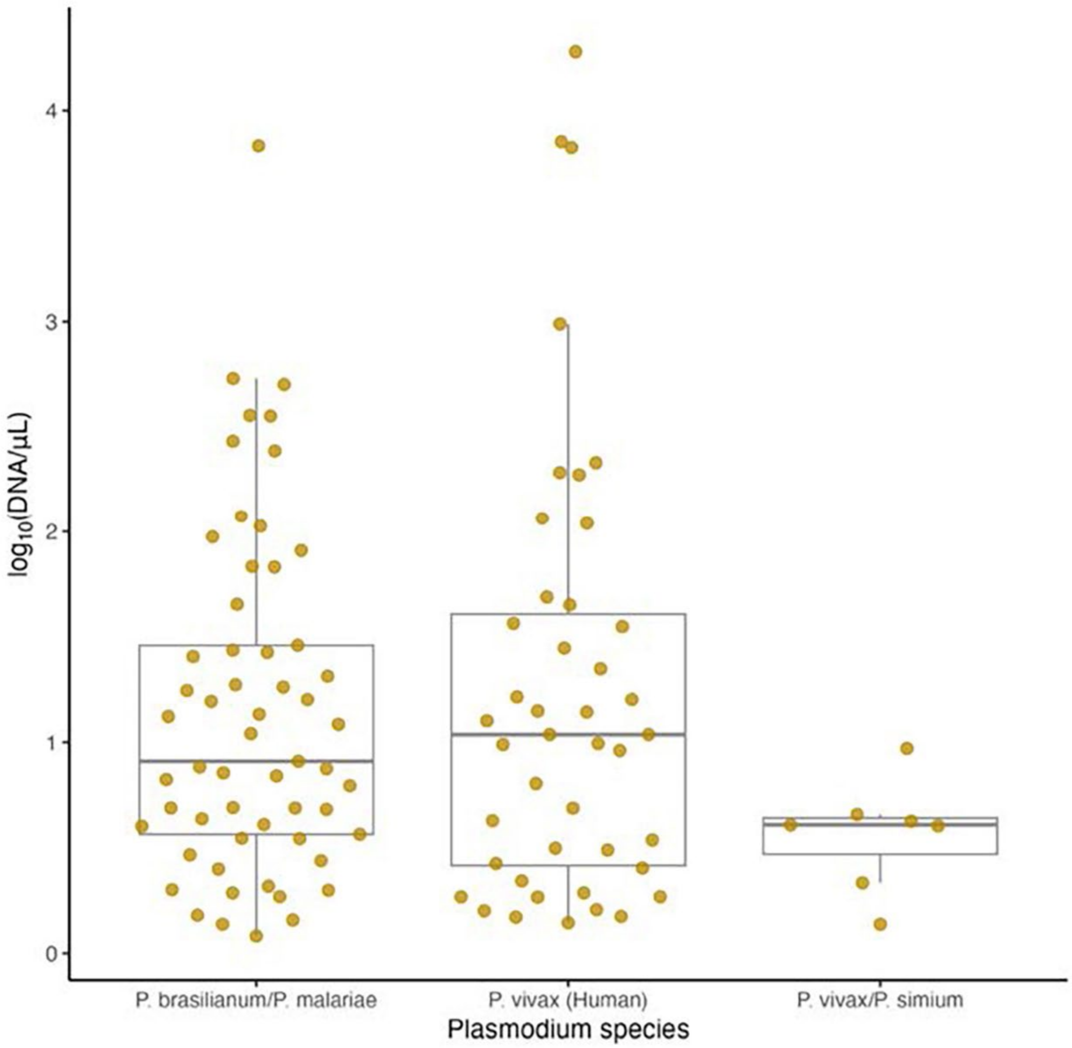

Appendix Figure 2. Supplementary Figure S2. Parasite load comparison between host groups. Comparison of parasite burden ($\log_{10}$[DNA/µL]) obtained by 18S qPCR in *P. vivax*/*P. simium* and *P. brasilianum*/*P. malariae* infections in humans (N = 43) and NHPs (N = 64).

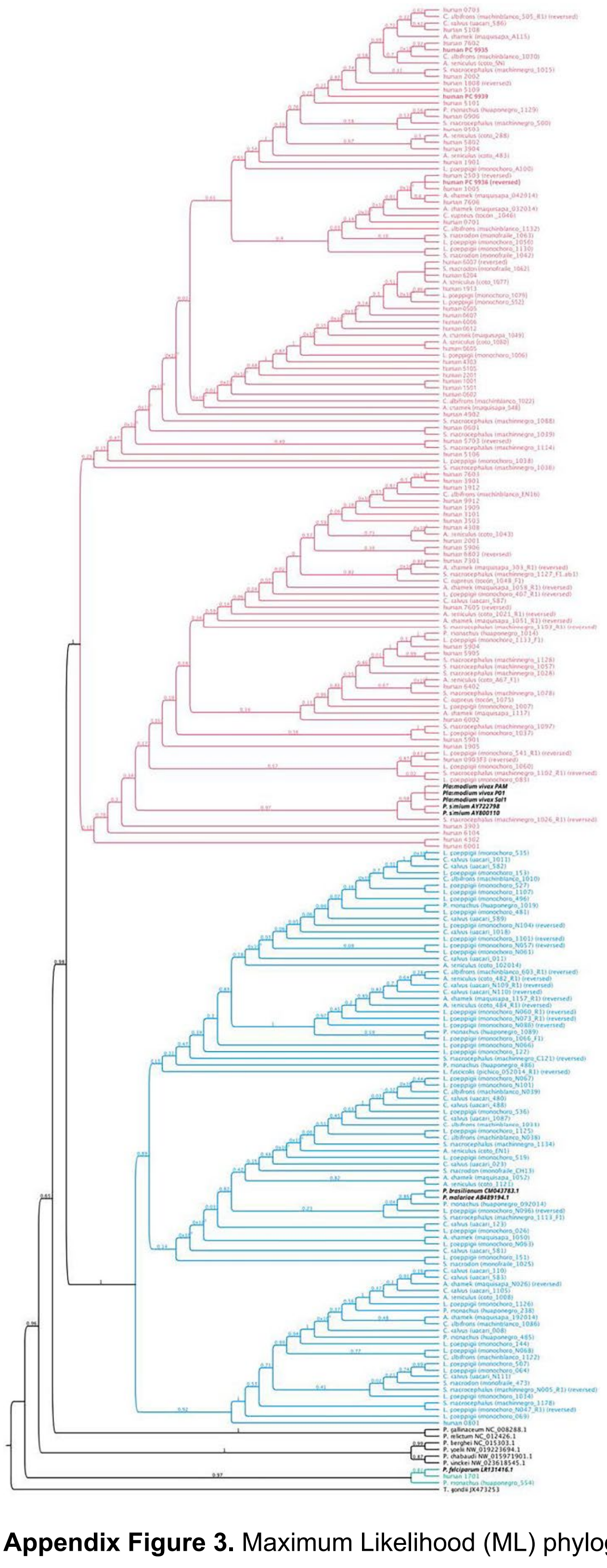

Appendix Figure 3. Maximum Likelihood (ML) phylogenetic tree of *Plasmodium* spp. ML tree (FastTree 2.1.11) based on ~776 bp of cytb sequences (n = 209). Clades colored: pink (Pv/Ps), blue (Pb/Pm), turquoise (Pf). Field researchers in bold; references in black.

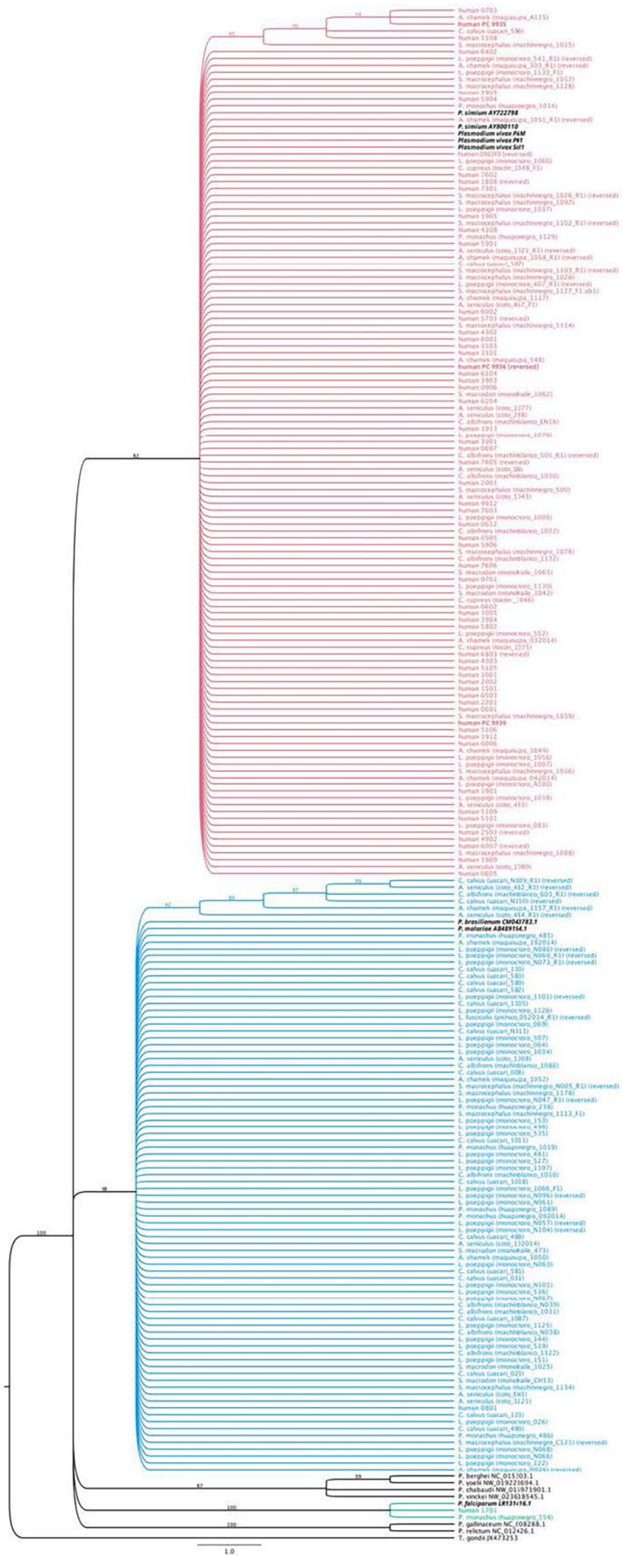

Appendix Figure 4. Neighbor-Joining (NJ) phylogenetic tree of *Plasmodium* spp. NJ tree with 1000 bootstrap replicates using human cytb sequences (n = 60) and NHPs (n = 149). Lineage colours and bold highlighting as above. Bootstrap ≥70% shown.

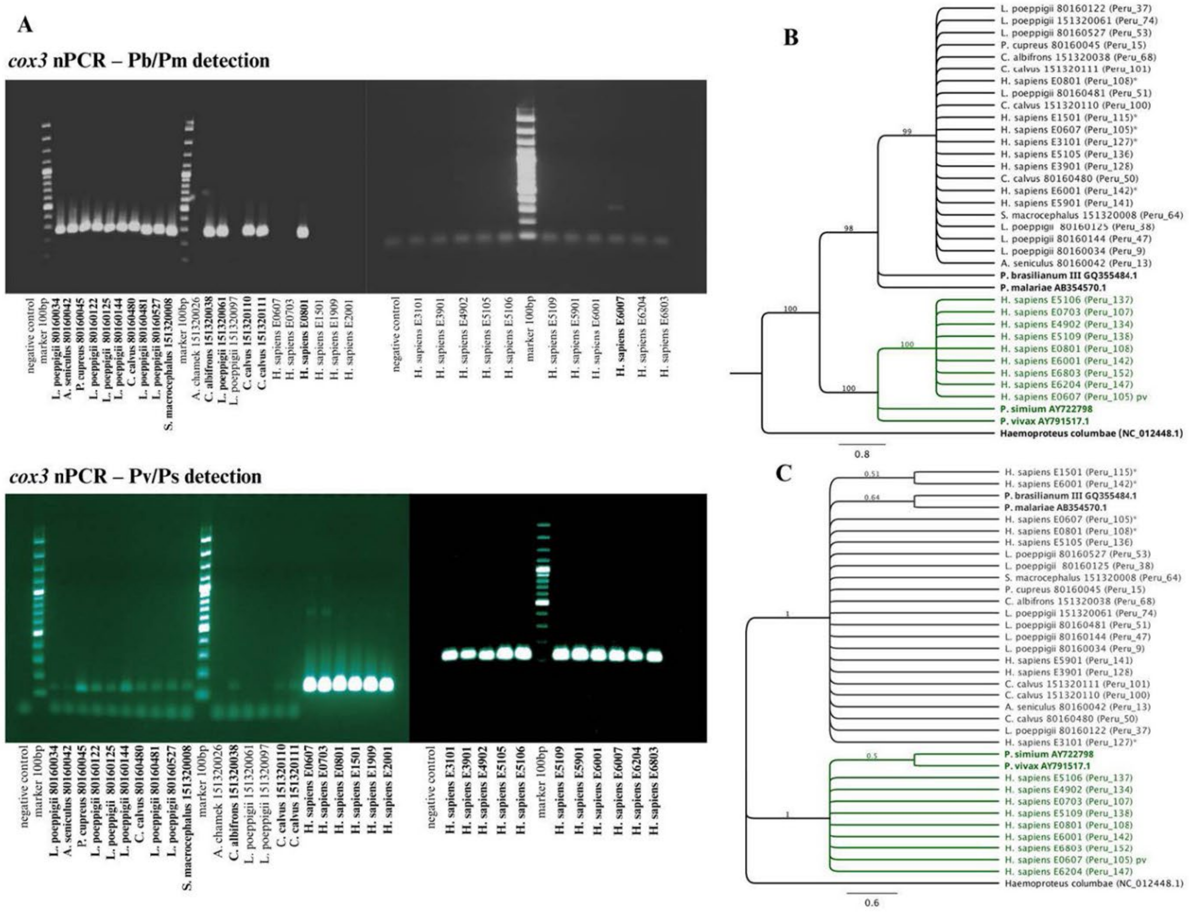

Appendix Figure 5. Validation of *Plasmodium* spp. detection by cox3 nPCR and phylogenetic analysis. (A) Gel electrophoresis of nested PCR products targeting the cox3 gene, showing representative bands for *P. brasilianum*/*P. malariae* (top panel) and *P. vivax*/*P. simium* (bottom panel) in non-human primates (NHPs) and humans. (B) Neighbor-joining (NJ) and (C) Bayesian inference (BI) phylogenetic trees based on cox3 sequences from NHP and human samples. The Pb/Pm clade is black, and the Pv/Ps clade is green. *Haemoproteus columbae* was used as an outgroup.

Reference

1. Isozumi R, Fukui M, Kaneko A, Chan CW, Kawamoto F, Kimura M. Improved detection of malaria cases in island settings of Vanuatu and Kenya by PCR that targets the *Plasmodium* mitochondrial cytochrome c oxidase III (*cox3*) gene. Parasitol Int. 2015;64:304–8. PubMed https://doi.org/10.1016/j.parint.2014.09.006
